# Role of the Demethylase AlkB Homolog H5 in the Promotion of Dentinogenesis

**DOI:** 10.3389/fphys.2022.923185

**Published:** 2022-06-15

**Authors:** Cheng Tian, Jihua Chai, Weidong Liu, Xinye Zhang, Yashu Li, Huanyan Zuo, Guohua Yuan, Haojian Zhang, Huan Liu, Zhi Chen

**Affiliations:** ^1^ The State Key Laboratory Breeding Base of Basic Sciences of Stomatology, Key Laboratory of Oral Biomedicine, Ministry of Education (Hubei-MOST KLOS & KLOBM), School and Hospital of Stomatology, Wuhan University, Wuhan, China; ^2^ Frontier Science Center for Immunology and Metabolism, Medical Research Institute, School of Medicine, Wuhan University, Wuhan, China; ^3^ Department of Periodontology, School and Hospital of Stomatology, Wuhan University, Wuhan, China; ^4^ Department of Cariology and Endodontics, School and Hospital of Stomatology, Wuhan University, Wuhan, China

**Keywords:** dentinogenesis, odontoblast, N6-methyladenosine, AlkB homolog H5, transcription factors

## Abstract

Dentinogenesis is a key process in tooth formation and is regulated by a series of pre- and post-transcriptional regulations. N6-methyl-adenosine (m^6^A), which is the most prevalent internal chemical modification that can be removed by the RNA demethylase AlkB homolog H5 (ALKBH5), has recently been reported to be involved in several biological processes. However, the exact function of ALKBH5-mediated m^6^A modification in tooth development remains unclear. Here, we showed that Alkbh5 was expressed in pre-odontoblasts, polarizing odontoblasts, and secretory odontoblasts. *Alkbh5* overexpression in the mouse dental papilla cell line mDPC6T promoted odontoblastic differentiation. Conditional knockout of *Alkbh5* in *Dmp1*-expressing odontoblasts led to a decrease in number of odontoblasts and increased pre-dentin formation. Mechanistically, RNA sequencing and m^6^A sequencing of *Alkbh5*-overexpressing mDPC6T cells revealed that *Alkbh5* promoted odontoblast differentiation by prolonging the half-life of *Runx2* transcripts in an m^6^A-dependent manner and by activating the phosphatidylinositol 3-kinase/protein kinase B pathway. Notably, the loss of Alkbh5 expression in odontoblasts impaired tertiary dentin formation *in vivo*. These results suggested that the RNA demethylase ALKBH5 plays a role in dentinogenesis.

## Highlights

• *Alkbh5* overexpression and knockdown regulate odontoblast differentiation.
• Loss of *Alkbh5* in odontoblasts *in vivo* impairs the formation of primary and tertiary dentin.
• ALKBH5-m^6^A regulates *Runx2* expression by affecting RNA degradation rate.
• *Alkbh5* promotes PI3K/AKT pathway activity to promote mouse dental papilla cell line odontoblastic differentiation.

## Introduction

Dentinogenesis is an important biological mineralization process regulated by several modifications including signaling pathways, transcriptional factors, and epigenetic modification of specific genes. Signaling pathways, such as bone morphogenetic protein, sonic hedgehog, Notch, and Wnt/catenin signaling, play a key role in tooth development (Jussila and Thesleff, 2012; Martín-González et al., 2019). Transcriptional factors including Kruppel-like factor 4 (KLF4), Runt-related transcription factor 2 (RUNX2; Cbfa1), and SP1 promote odontoblast differentiation (Sun et al., 2019; Tao et al., 2019; Lin et al., 2021). During cell differentiation, specific gene expression patterns may be regulated by epigenetic factors such as histone and DNA modifications. Previous studies have shown that the p300-and histone deacetylase 3-mediated upregulation of histone acetylation regulates odontoblast differentiation (Tao et al., 2020); KLF4 regulates the transcription of dentin matrix acidic phosphoprotein 1 (*Dmp1*) *and Sp7 via* histone acetylation, which is essential for dentinogenesis (Tao et al., 2019); During the odontoblastic differentiation of dental pulp cells, SP1 regulates KLF4 function *via* the SP1-binding motif controlled by DNA methylation (Sun et al., 2019). However, the role of RNA modification during odontoblast differentiation is not fully understood.

N6-methyladenosine (m^6^A), which represents the most common chemical modification of mRNA, is added at the adenosine N6 position in eukaryotes (Zhou et al., 2016; Amort et al., 2017). The formation or removal of the m^6^A modification is catalyzed by methyltransferases (Jia et al., 2013; Zhang et al., 2016). The methyltransferase-like 3 (METTL3)-centered methyltransferase complex catalyzes the addition of the m^6^A modification, and α-ketoglutarate-dependent dioxygenase alkB homolog 5 (ALKBH5) or fat mass and obesity-associated protein catalyzes the removal from RNA (Punekar et al., 2013; Wang et al., 2016). These two enzymes can maintain the levels of RNA methylation modification in a steady state. m^6^A modification affects various aspects of mRNA, including pre-mRNA splicing, alternative polyadenylation, RNA stability, and translation (Jia et al., 2013; Wang X. et al., 2014; Liu et al., 2015; Zhang et al., 2016). METTL3 plays an important role in determining the lineage of mesenchymal stem cells by regulating the translation of parathyroid hormone receptor 1 (Wu et al., 2018); *METTL3* knockdown downregulates nuclear factor I C and inhibits odontogenesis in dental roots (Sheng et al., 2021). The ALKBH5-protein arginine N-methyltransferase six pathway affects the osteogenic differentiation of bone marrow mesenchymal stem cells (Li et al., 2021). ALKBH5 promotes osteoblast differentiation by regulating the stability of *RUNX2* mRNA (Feng et al., 2021). Based on these findings, we hypothesized that m^6^A methylation may be involved in odontoblast differentiation.

In the present study, ALKBH5 expression during odontoblast differentiation was confirmed. Conditional knockout of *ALKBH5* in odontoblasts led to a decline in the number of odontoblasts and increase in pre-dentin width. Mechanistically, we revealed that the m^6^A demethylase ALKBH5 plays a positive role in regulating the odontoblast differentiation of a dental papilla cell line (mDPC6T) by decreasing the mRNA decay rate of *Runx2* and activating the phosphatidylinositol 3-kinase/protein kinase B (PI3K/AKT) pathway based on RNA sequencing (RNA-seq) and m^6^A sequencing (m^6^A-seq) analysis *in vitro*. Further, the loss of ALKBH5 in odontoblasts *in vivo* impaired the formation of tertiary dentin.

## Materials and Methods

### Animal Maintenance

Institutional Animal Care and Use Committees at the School and Hospital of Stomatology of Wuhan University approved to conduct this experimental protocol (protocol no. S07920090B). Kunming mice were obtained from the Hubei Provincial Center for Disease Control and Prevention (Hubei CDC). C57BL/6J (CD45.2) background *Alkbh5* ^fl/fl^ mice and dentin matrix protein 1 (*Dmp1*)-cre mice were obtained as a gift from Haojian Zhang Laboratory, respectively. *Dmp1*-Cre mice and *Alkbh5* ^fl/fl^ mice were produced by Bcgen (Beijing Biocytogen Co., Ltd.). To generate odontoblast-conditional *Alkbh5* knockout mice, we crossed *Dmp1*-Cre mice with *Alkbh5* ^fl/fl^ mice to obtain *Dmp1*-Cre; *Alkbh5* ^fl/+^ mice. By copulation *Dmp1*-Cre; *Alkbh5* ^fl/+^ male mice with *Alkbh5* ^fl/fl^ female mice, we got *Dmp1*-Cre; *Alkbh5* ^fl/fl^ mice as a conditional homozygous *Alkbh5*-knockout mice. The *Alkbh5* ^fl/fl^ littermates were used as the control. The genotype of these transgenic mice was determined by extracting the tail genomic DNA for PCR analysis. Genotyping of *Alkbh5* ^fl/fl^ mice primers included the following: F-primer, 5′-GAG​TGA​CAA​TGG​AAA​TCA​CCA​GGG​T-3′, and R-primer, 5′-GGA​TGA​AGC​CTC​ATC​AGG​AGA​ACA​GT-3′. The endogenous product size of *Alkbh5* was 342 bp, and the product size of *Alkbh5* ^fl/fl^ was 414 bp. Genotyping of *Dmp1*-Cre mice primers included the following: primer-1, 5′-TGG​AAG​CTG​ACA​GTA​GGA​AAC-3′, primer-2, 5′-TGACATCATCCCACGTACTTAAGC-3′and primer-3, 5′- TGG​TGC​ACA​GTC​AGC​AGG​TTG-3′. The product size of hemizygous for *Dmp*1-Cre was 208 bp, and the product size of endogenous *Dmp1* was 334bp.

### Cell Culture and Differentiation

Our group has constructed a dental papilla cell line (extraction of mandibular molar from E16.5 of Kuming Mice), named mDPC6T, that retains most of the properties and features of primary mouse dental papilla cells (Lin et al., 2013a). To induce mDPC6T odontoblast differentiation, cells were cultured with 10 mmol/L of sodium β-glycerophosphate (Sigma, St Louis, MO, United States), 50 µg/ml of ascorbic acid (Sigma, St Louis, MO, United States), and 10 nmol/L of dexamethasone (Sigma, St Louis, MO, United States) for 0,5,7,11 and 14 days. Cells were collected at different time points for further experiment.

### Transfection

mDPC6T cells were transfected with a negative siRNA and si*Alkbh5* (Genecreate Biological Engineering Co., Ltd., Wuhan, China) at a final concentration of 100 nM. Lipofectamine 2000 (Thermo Fisher Scientific, Waltham, MA, United States) was applied according to the instructions. The sequences are shown in Supplementary Table S1. mDPC6T cells were cultures in odontoblast induction medium for 3 days after transfection.

The overexpression lentivirus was constructed, designed and cloned by Genechem Co., Ltd. Technology (Shanghai, China). Stable clones were sustained with 900 μg/ml Geneticin Selective Antibiotics (G418 Sulfate). The counts were observed by fluorescence microscopy to confirm that the transfection rate, and the overexpression results were detected by western blot assay. mDPC6T cells were cultures in odontoblast induction medium for 7 and 14 days after transfection.

MedChemExpress provided LY294002 and SC79. mDPC6T cells were added with 10 μM LY294002 after lentivirus transfection. mDPC6T cells were added with 10 μM SC79 after small interfering RNA (si*Alkbh5*) transfection.

### Alizarin Red S Staining

mDPC6T cells were induced in odontoblast induction medium and collected at different time point. mDPC6T were irrigation with PBS and fixation in 95% ethyl alcohol for 10 min, 1% alizarin red S (Sigma) solution. Nodules were recorded by an inverted phase-contrast microscope (Axiovert 40; Zeiss, Jena, Germany).

### Alkaline Phosphatase Activity Assay

Cells were cultured and lysed with 1% Triton X-100. ALP assay kit (Nanjing Jiancheng Bioengineering Institute, Nanjing, China) was utilized using standard methods.

### Quantitative Real Time Polymerase Chain Reaction

Total RNA from mDPC6T was extracted using HP Total RNA Kit (Omega Bio-tek, Norcross, GA, United States). RNA samples were reverse-transcribed for cDNA synthesis using HiScript^®^ II Q RT SuperMix for qPCR r222 (Vazyme Biotech Co., Ltd., Nanjing, China). qRT-PCR was performed using the HiScript^®^ II One Step qRT-PCR SYBR Green Kit by the CFX Connect™ Real-Time System (1855201, Bio-Rad, Hercules, CA, United States). *Gapdh* was utilized as an internal control. The 2−!!ΔCT methods were used to analyse the genes expression. The primers were synthesized by Sangon Biotech Co., Ltd. (Shanghai, China), and the sequences were shown in Supplementary Table S2.

### Western Blot Analysis

Total proteins obtained from the fraction of cell lysate and measured using BCA Protein Assay Kit (Thermo Fisher Scientific, Waltham, MA, United States of America). Protein was electrophoresed and then transfered membranel. Membranes were blocked and then incubated with the following primary antibodies: METTL3 (15073-1-AP, 1:1,000; Proteintech, Chicago, IL, United States of America), FTO (27226-1-AP, 1:1,000; Proteintech, Chicago, IL, United States of America), ALKBH5 (16837-1-AP, 1:1,000; Proteintech, Chicago, IL, United States of America), RUNX2 (12556S, 1:1,000, Cell Signaling Technology, Beverly, MA, United States of America), AKT (#9272, 1:1,000; Cell Signaling Technology, Boston, MA, United States of America) and P-AKT (#4060, 1:1,000; Cell Signaling Technology, Boston, MA, United States of America), DMP1 (A16832, 1:1,000; ABclonal, Wuhan, China), DSPP (NBP2-92546, 1:1,000, Novus Biologicals, Centennial, CO, United States of America), and β-ACTIN (PMK081Y, 1:8,000, BioPM, Wuhan, China). The membrane was then incubated with secondary antibodies (Abcam, Cambridge, UK). The protein binding developed with WesternBrightTM ECL solution (Advansta, San Jose, CA, United States of America). β-ACTIN was utilized as an internal control. ImageJ software was used for analyzing the band intensities.

### Micro-Computed Tomography and Histomorphometric Analyses

The mandibles of *Alkbh5* ^fl/fl^ and *Dmp1*-Cre; *Alkbh5* ^fl/fl^ mice were dissected for analysis. µCT was used to analyze the first molar. The width and length of the proximal mesial root of the first molar and the length of the whole tooth was measured analyzed by laminar sectioning (n > 6 mice for each group).

### Histology (HE) and Immunohistochemistry

Murine mandibular samples were dissected and fixed individually in 4% paraformaldehyde (PFA). 10% ethylenediaminetetraacetic acid (EDTA) was used for decalcified for several days. Next, samples were dehydration and paraffin embedding for histological analysis. Hematoxylin and eosin (HE) staining was performed using standard methods. Samples were treated with pepsin solution and incubated with ALKBH5 (1:200; Proteintech, Chicago, IL, United States ) antibodies for immunohistochemical staining. Diaminobenzidine reagent kit (Maixin, Fuzhou, China) was utilized to visualization after incubation with a horseradish peroxidase (HRP) secondary antibody and hematoxylin redyeing were counterstained. Tissue sections were recorded using a digital pathology section scanner (LUMENCOR, United States of America).

### Immunofluorescence

Samples were blocked with 2.5% bovine serum albumin (BSA) and incubated with primary antibody ALKBH5/Runx2 (12556S, 1:100, Cell Signaling Technology, Beverly, MA, United States) and KI67 (1: 200; Abcam, Cambridge, UK). After incubation with fluorescent secondary antibodies (Jackson Immuno Research, West Grove, PA, United States), nuclear were stained with 4′,6-diamidino-2-phenylindole (DAPI). Tissue sections were recorded using a digital pathology section scanner (LUMENCOR, United States).

### Acid-Etching SEM

The mandibles of 14 days postnatal mice were first fixed in a solution containing 2.5% glutaraldehyde (pH 7.4), followed by polishing of the dentin surface, 30% phosphoric acid treatment for 20 s, and then transferred to 5.25% sodium hypochlorite and saline for alternate ultrasonic cleaning. The surface of dentin was examined using an SEM microscope (TESCAN, Brno, Kohoutovice Czech Republic).

### TUNEL Staining

TUNEL BrightRed Apoptosis Detection Kit (Vazyme Biotech Co., Ltd., Nanjing China) was utilized for detecting apoptosis, following the manufacturer’s instruction. Tissue sections were photographed using a digital pathology section scanner (LUMENCOR, United States).

### RNA-Seq and m^6^A-Seq and Data Analysis

For RNA-seq, poly (A) mRNA was purified from total RNA. The DNA libraries were prepaired and sequenced on an Illumina Hiseq X Ten platform (Novogene Bioinformatics Technology Co. Ltd.). Gene levels were quantified with Kallisto (v0.43.1) using tximport (v1.10.1) to summarize RNA-seq. Data normalization and differential expression analysis were performed using EdgeR (v3.24.3).

For m^6^A-seq, poly (A) mRNA was purified and fragmented. The fragments were incubated with affinity-purified anti-m^6^A polyclonal antibody (Synaptic Systems, Germany) followed by immunoprecipitation with protein-A beads (Thermo Fisher, 21,348). The fragments were subjected to library construction with TruSeq Stranded mRNA Library Prep Kit (Illumina, San Diego, CA) and sequenced on an Illumina HiSeq X Ten (Novogene Bioinformatics Technology Co. Ltd.). The above two sequencing databases were read together and matched with the HISAT2 (v2.1.0) mm10 mouse reference genome. ExomePeak (v2.16.0) calls m^6^A peaks. Deeptools (v2.0) generates m^6^A-seq data. The DAVID tool (v6.8) was used for functional enrichment analysis and clusterProfiler (v3.10.1) was used to visualize it (Huang et al., 2009; Yu et al., 2012). IGV (v2.4) was used for m^6^A peak visualization at the same scale. We have deposited the sequencing data in a public, community-supported repository: The BIG Submission. The accession number is: CRA006723.

### m^6^A-qRT-PCR

RNA was processed as mentioned above in m^6^A-seq. The relative enrichment was normalized to the input and data from m^6^A immunoprecipitation samples were analyzed.

### RNA Decay Assay

mDPC6T cells transfected with *Alkbh5* overexpression lentivirus were treated by odontoblast induction medium and cultured for 3 days. Subsequently, RNA at 0, 1, 2, 4, 6 and 8 h after addition of 5 μg/ml actinomycin D (ActD, MedChemExpress) was extracted and detected by qRT-PCR as described above.

### EdU (5-Ethynyl-2-deoxyuridine) Cell Proliferation Assay

Cells were incubated with odontoblast induction medium for 5 days, then were treated with 50 uM EdU for 3 h. EdU Imaging Kit (Cy3) (APE*BIO Technology, Houston, United States) was utilized for incubating EdU according to the manufacturer’s instructions and observed by a fluorescence microscope (Leica, Wetzlar, Germany).

### Tertiary Dentin Formation Model

Mice were divided into control drilling groups and *Alkbh5-cKO* drilling groups. At 6 weeks of age, eight mice per group (bilateral model) were used to detect the formation of tertiary dentin. A 0.1 mm pore-shaped cavity was prepared on the proximal mesial surface of the upper first molar *via* a turbine with spherical burs (diameter, 0.5 mm) (Saito et al., 2013). Tissues were collected at 14th day after the operation.

### Statistical Analysis

A *p*-value < 0.05 was considered to be statistically significant. Differences between groups were tested by Student’s *t* test or one-way ANOVA. All quantitative data are expressed as mean ± standard deviation (SD), with at least three independent samples and sample sizes were labeled for each legend.

## Results

### *Alkbh5* Was Upregulated During Odontoblast Differentiation *in Vivo* and *in Vitro*

**FIGURE 1:**
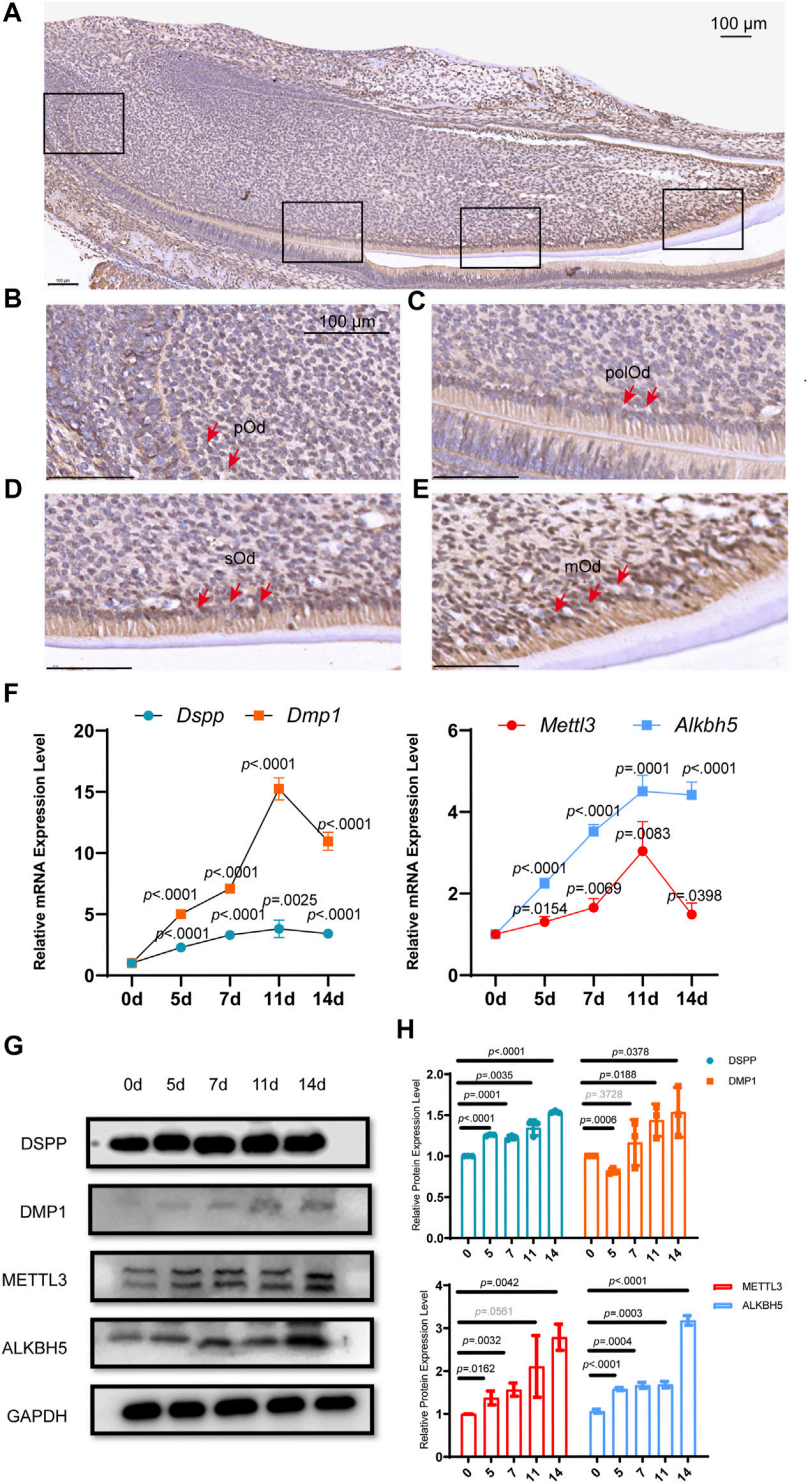
*Alkbh5* was upregulated during mouse odontoblast differentiation *in vivo* and *in vitro*. **(A)** Immunohistochemistry analysis of the expression of ALKBH5 in the PN2.5 murine mandibular incisors. ALKBH5 protein expression was low in **(B)** pOd and was strongly expressed in **(C)** polOd **(D)** sOd, and **(E)** mOd. **(F)** The odontoblast markers *Dspp* and *Dmp1*, and m^6^A methylase *Mettl3* and demethylase *Alkbh5* were upregulated after induction. **(G)** The protein expression levels of METTL3, ALKBH5, DSPP, and DMP1 were significantly increased. **(H)** Quantification of the DMP1, DSPP, METTL3, and ALKBH5 protein expression level during odontoblast induction. **(A–E)** Scale bar: 100 μm Alkbh5, AlkB homolog H5; pOd, pre-odontoblasts; polOd, polarizing odontoblasts; sOd, secretory odontoblasts; mOd, mature odontoblasts; Dspp, dentin sialophosphoprotein; Dmp1, dentin matrix acidic phosphoprotein 1; Mettl3, methyltransferase-like 3; PN2.5, postnatal day 2.5.

Immunohistochemistry was performed using postnatal day 2.5 (PN2.5) mouse mandibular incisors to detect the expression of ALKBH5 during odontoblast differentiation *in vivo* (Figure 1A). ALKBH5 expression was low in pre-odontoblasts and polarizing odontoblasts (Figures 1B,C), but an increased-level expression in secretory odontoblasts (Figure 1D) and was considerably high in mature odontoblasts (Figure 1E).

Dental papilla cells can differentiate into odontoblast-like cells, which are precursors of odontoblasts *in vitro* and synthesize pre-dentin-dentin components (Bègue-Kirn et al., 1998; Tziafas and Kodonas, 2010). Since primary cultured dental papilla cells have a high variability and short lifespan, we established a mouse dental papilla immortalized cell line named mDPC6T (Lin et al., 2013b). To evaluate the expression pattern of *Alkbh5* during odontoblast differentiation *in vitro*, mDPC6T cells were cultured in odontoblast induction medium for 0, 5, 7, 11, and 14 days. The odontoblast markers *Dmp1* and dentin sialophosphoprotein (*Dspp*) were upregulated during odontoblast induction based on quantitative reverse transcription-polymerase chain reaction (qRT-PCR) (Figure 1F) and western blotting (Figure 1G). Quantification of the relative protein expression levels is shown in Figure 1H. During odontoblast differentiation of mDPC6T cells, mineralized nodules were detected after induction (Supplementary Figure S1), and an increased deposition of mineralized matrix was observed after 14 days of odontoblast differentiation that further confirmed the odontoblast differentiation of mDPC6T cells. To evaluate the role of m^6^A modification in odontoblast differentiation of mDPC6T cells, the expression levels of an m^6^A methyltransferase (*Mettl3*) and demethylase (*Alkbh5*) were analyzed during the odontoblast induction of mDPC6T cells. Both the mRNA and protein levels of *Alkbh5* increased after odontoblast differentiation (Figures 1F–H). The expression level of ALKBH5 during odontoblast differentiation was in accordance with the expression levels of odontoblast markers. These results were also in agreement with the upregulation of *Alkbh5* during odontoblast differentiation *in vivo* (Figures 1A–E).

### *Alkbh5* Improved Odontoblast Differentiation of mDPC6T Cells

**FIGURE 2:**
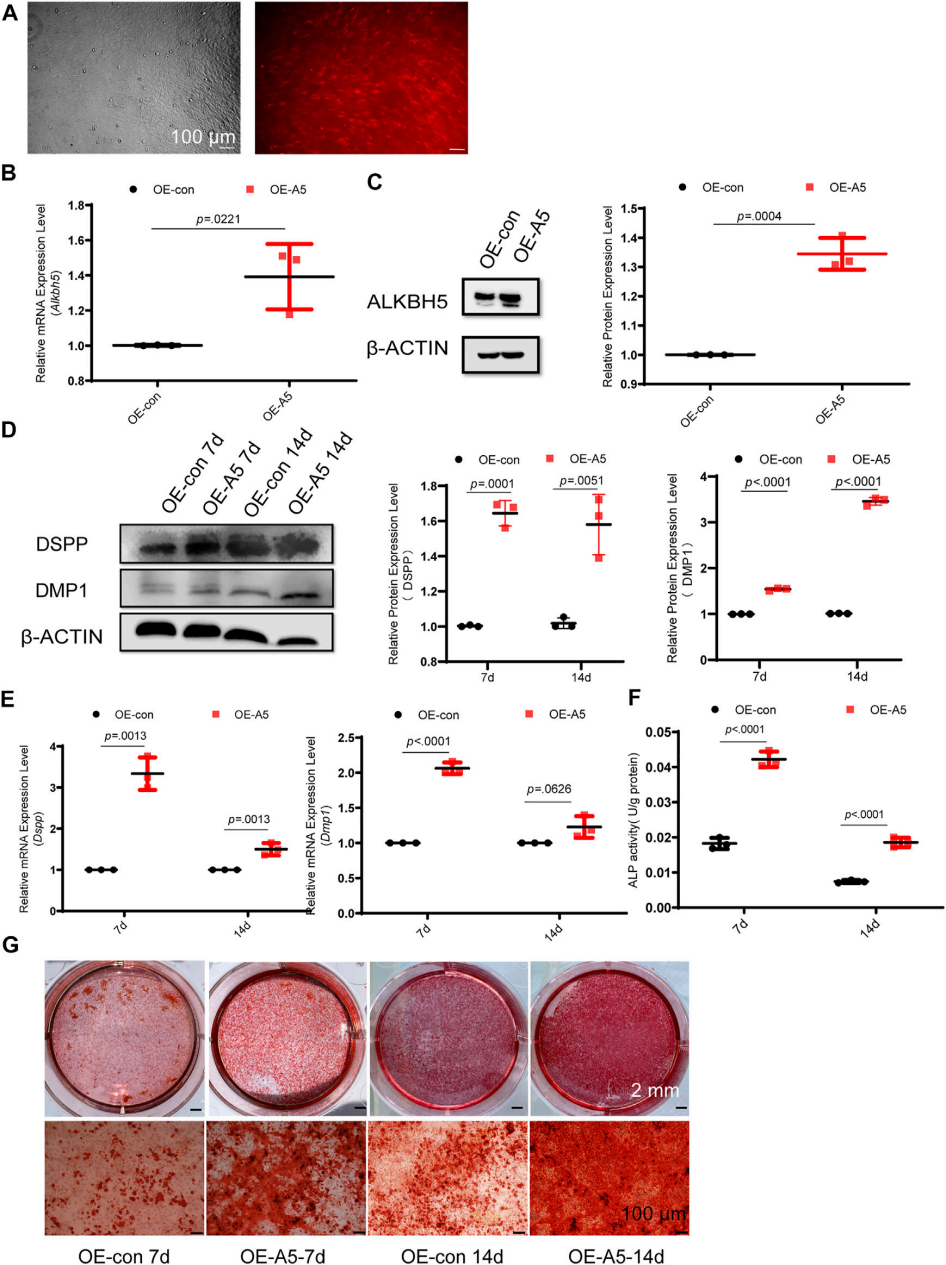
*Alkbh5* improved odontoblast differentiation of mDPC6T cells. **(A)** Transfer efficiency of overexpressed *Alkbh5* in mDPC6T cells was detected and observed *via* red fluorescent protein labeling under a microscope (see above) after transfection for 72 h. The lower panel shows the immunofluorescence images taken simultaneously. Scale bar: 100 μm (original magnification × 100). **(B,C)** *Alkbh5* expression level was measured *via* qPCR and western blotting in the *Alkbh5* overexpression group and negative control group. **(D)** The protein levels of DSPP and DMP1 in the *Alkbh5* overexpression group and control group were measured *via* western blotting after 7 and 14 days of odontoblast induction. **(E)** The mRNA expression levels of *Dspp* and *Dmp1* were measured *via* qRT-PCR after 7 and 14 days of odontoblast induction. **(F)** ALP activity was determined. Alkbh5, AlkB homolog H5; Dspp, dentin sialophosphoprotein; Dmp1, dentin matrix acidic phosphoprotein 1; qRT-PCR, quantitative reverse transcription-polymerase chain reaction; ALP, alkaline phosphatase. **(G)** Mineralized nodule formation was analyzed on days 7 and 14 *via* Alizarin Red S staining in the *Alkbh5* overexpression groups and the control group undergoing odontoblast induction. Scale bars: 2 mm and 100 μm (high magnification).

To investigate the role of Alkbh5 in odontoblast differentiation *in vitro*, we used a lentivirus-mediated approach to overexpress *Alkbh5* in mDPC6T cells. Lentiviral gene transfer efficiency in mDPC6T cells was measured based on the proportion of fluorescent cells. Transfer efficiency reached 90% within 48 h (Figure 2A). *Alkbh5* overexpression was confirmed *via* qRT-PCR and western blotting analyses (Figures 2B,C). *Alkbh5* mRNA and protein expression levels increased to 150% in the *Alkbh5* overexpression group relative to that in the control group, suggesting that *Alkbh5* was effectively overexpressed in mDPC6T cells. Next, we investigated the effect of *Alkbh5* overexpression on odontoblast differentiation based on the expression levels of *Dmp1* and *Dspp*. *Dmp1* and *Dspp* were markedly upregulated in the *Alkbh5* overexpression group (Figures 2D,E). Alkaline phosphatase was upregulated in the *Alkbh5* overexpression group (Figure 2F). In addition, we observed enhanced matrix mineralization in the *Alkbh5* overexpression group after induction (Figure 2G). These results suggested that Alkbh5 promoted odontoblast differentiation.

*Alkbh5* small interfering RNA (si*Alkbh5*) or a control siRNA were transfected into mDPC6T cells. The #1 and #2 *siAlkbh5* groups exhibited 60% *Alkbh5* expression levels relative to that in the control group (Supplementary Figures S2A, B). Therefore, transfection with the #1 and #2 *siAlkbh5* siRNAs created the best knockdown effect and were used in the following experiments. The recombinant si*Alkbh5* and control siRNA*-*transfected mDPC6T cells were treated with odontoblast differentiation induction medium for 3 days. Western blotting and qRT-PCR analyses showed that Dspp and Dmp1 expression levels were lower in the si*Alkbh5* group compared with those in the control siRNA group (Supplementary Figures S2C, D). Furthermore, the si*Alkbh5* group showed attenuated matrix mineralization after induction (Supplementary Figure S2E). These data emphasize the importance of *Alkbh5* upregulation in subsequent odontoblast differentiation *in vivo*.

### Impaired Primary Dentin Formation in *Dmp1*-Cre; *Alkbh5* ^*fl/fl*^ Mice

**FIGURE 3:**
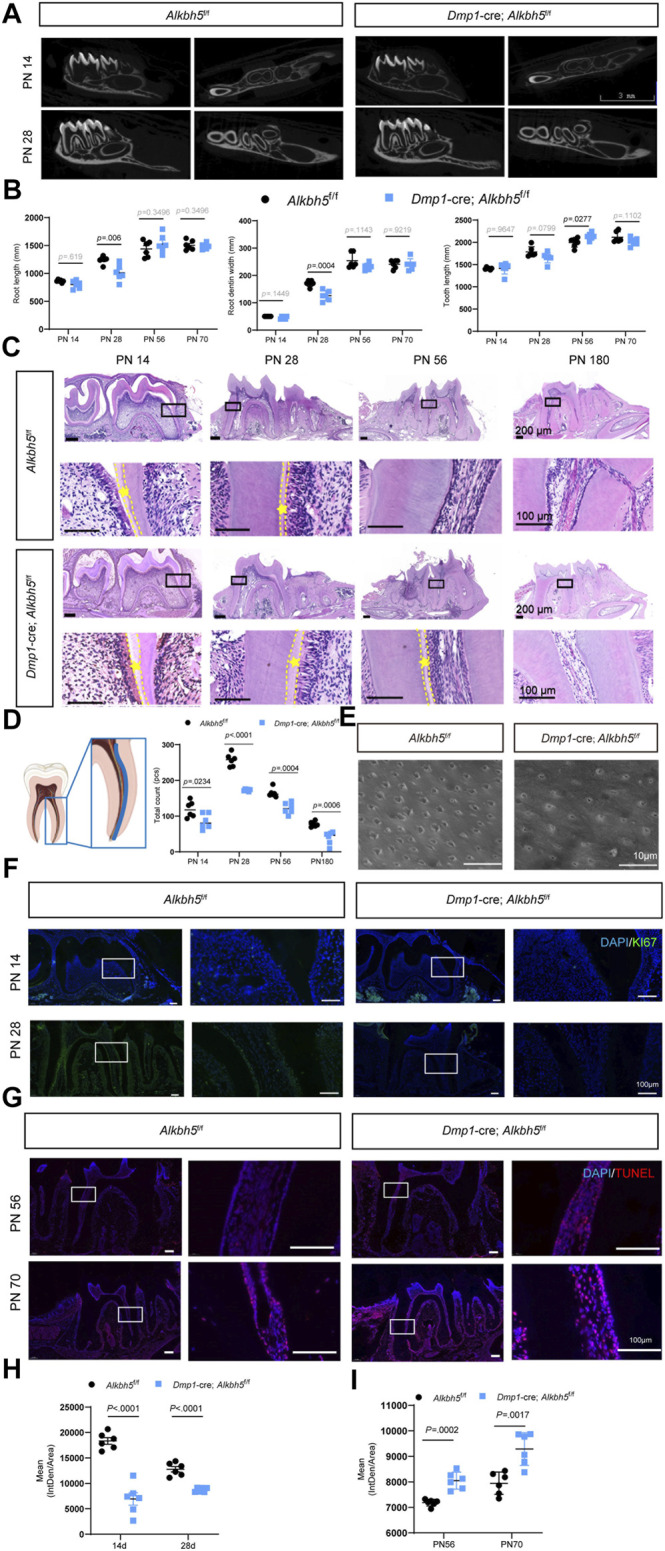
Impaired primary dentin formation in *Dmp1*-Cre; *Alkbh5* ^*fl/fl*^ mice. **(A)** Structural μCT imaging of the mandibular first molars derived from *Dmp1*-Cre; *Alkbh5* ^*fl/fl*^ and *Alkbh5* ^*fl/fl*^ mice at PN 14 and PN 28. Scale bar: 3 mm. **(B)** Quantitative μCT analyses of root dentin width, root length, and tooth length at PN 14, PN 28, PN 56, and PN 70 from *Dmp1*-Cre; *Alkbh5* ^*fl/fl*^ and *Alkbh5* ^*fl/fl*^ mice. **(C)** Representative H&E staining of the first molar structure in the mandibles for *Dmp1*-Cre; *Alkbh5* ^*fl/fl*^ and *Alkbh5* ^*fl/fl*^ mice at PN 14, PN 28, PN 56, and PN 180. The area between the dotted lines represents the pre-dentin (asterisk *) area. Scale bar: 200 and 100 μm (high magnification). **(D)** Number of odontoblasts determined from H&E-stained sections. The analysis area was set in the mandibular first molar mesial odontoblasts from the furcation to the apical foramen in the area of the blue lines (left). **(E)** Representative acid-etched scanning electron microscopy images of radicular odontoblast process. Scale bar: 10 μm. **(F)** Immunofluorescence analysis of the expression of KI67 (green) in *Dmp1*-Cre; *Alkbh5* ^*fl/fl*^ and *Alkbh5* ^*fl/fl*^ mice at PN 14 and PN 28. **(G)** *in situ* TUNEL assay (red) analysis of the apoptosis signals for *Dmp1*-Cre; *Alkbh5* ^*fl/fl*^ and *Alkbh5* ^*fl/fl*^ mice at PN 56 and PN 70. **(H)** Quantitative analyses of the expression of KI67 in *Dmp1*-Cre; *Alkbh5* ^*fl/fl*^ and *Alkbh5* ^*fl/fl*^ mice at PN 14, and PN 28. Scale bar: 100 μm. **(I)** Quantitative analyses of the expression of TUNEL apoptosis signals for *Dmp1*-Cre; *Alkbh5* ^*fl/fl*^ and *Alkbh5* ^*fl/fl*^ mice at PN 56 and PN 70. Scale bar: 100 μm μCT, micro-computed tomography; Alkbh5, AlkB homolog H5; Dmp1, dentin matrix acidic phosphoprotein 1; H&E, hematoxylin and eosin; TUNEL, terminal deoxynucleotidyl transferase (TdT) dUTP Nick-End Labeling; PN, postnatal day.

To determine the function of *Alkbh5* in dentin formation, *Alkbh5* was knocked out from odontoblasts by cross-breeding mice with *Dmp1*-Cre mice to establish *Dmp1*-Cre; *Alkbh5* ^*fl/fl*^ mice (hereafter as *Alkbh5-cKO*). *Dmp1* is an important regulator in odontoblast differentiation and is expressed by functional and fully differentiated odontoblasts (Balic and Mina, 2011; Vijaykumar et al., 2019; Isono et al., 2021). Firstly, we performed morphological analysis on the mandibular first molars of *Alkbh5-cKO* mice; *Alkbh5* ^fl/fl^ mice. No significant difference in the root dentin width and length of the mandibular first molar *via* μCT reconstruction of the mandible in *Alkbh5-cKO* mice compared to *Alkbh5* ^fl/fl^ control littermates (Figure 3A, Supplementary Figure S3). Although the phenotype is not striking, quantitative analyses showed that root length, root dentin width in *Alkbh5-cKO* mice were reduced at PN 28 compared to *Alkbh5* ^fl/fl^ littermates (Figure 3B). To observe the morphology and quantity of odontoblasts and the formation of dentin, hematoxylin and eosin staining of the mandibular first molar was performed. The number of odontoblasts was found to be reduced and a thickened pre-dentin layer was observed in *Dmp1*-Cre; *Alkbh5* ^fl/fl^ mice (Figure 3C). The number of odontoblasts was counted from the root furcation to the apical foramen of the first mandibular molar (Figure 3D). Quantitative analysis showed that the number of terminal odontoblasts decreased drastically (Figure 3D).

To further evaluate the odontoblast processes, we performed scanning electron microscopy using acid-etched samples to enhance visualization of dentin tubules. We found a reduced density of odontoblast-related processes in *Alkbh5-cKO* mice (Figure 3E). Based on a considerable decrease in number of odontoblasts, we evaluated cell proliferation and activation of apoptosis. The *Dmp1*-Cre; *Alkbh5* ^fl/fl^ mice showed weaker positive signals for KI67 *via* immunofluorescence staining at PN 14 and PN 28 (Figures 3F–I) and more strong positive signals based on an *in situ* TUNEL assay at PN 56 and PN 70 compared to those in control mice (Figures 3G,I). These data further suggest that the deletion of *Alkbh5* in terminal odontoblasts decreased the number of odontoblasts and affected the thickness of the pre-dentin layer by regulating proliferation and apoptosis in terminal odontoblasts *in vivo*, which eventually resulted in dysfunctional primary dentinogenesis.

### Whole-Transcriptome m^6^A-Seq and RNA-Seq of *Alkbh5* Downstream Regulatory Genes

**FIGURE 4:**
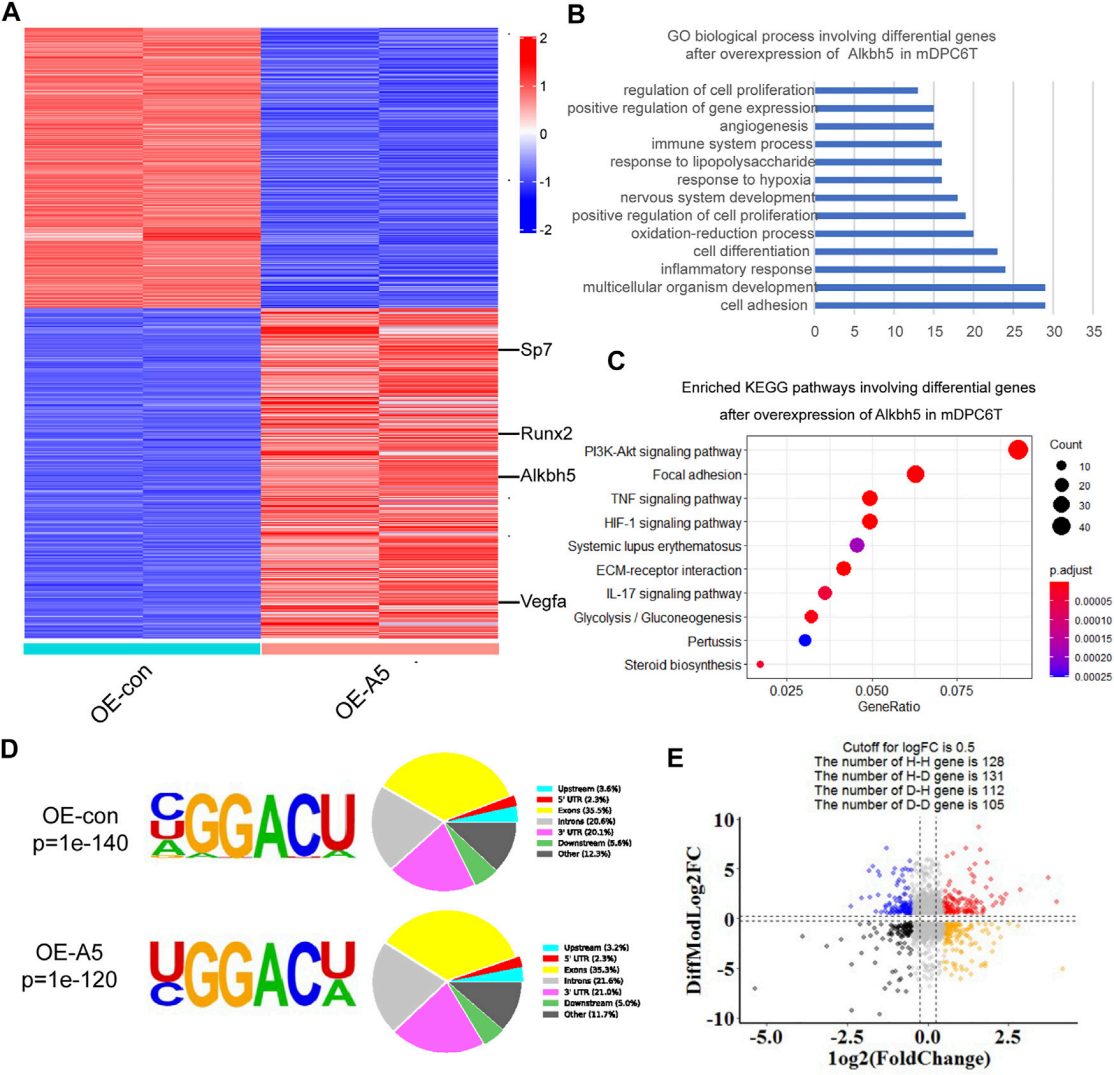
Whole-transcriptome m^6^A-seq and RNA-seq analysis of *Alkbh5* downstream regulatory genes. **(A)** Heat map shows the differentially expressed genes related to *Alkbh5* in mDPC6T cells. **(B,C)** GO and KEGG enrichment analyses of differentially expressed genes in *Alkbh5*-overexpressing mDPC6T cells. **(D)** Specific motif sequences of the *Alkbh5* overexpression and control groups. Portions of the m^6^A peak are distributed in upstream, 5′-UTR, Exon, Introns, Downstream, and 3′-UTR across or in other regions of the entire set of mRNA transcripts. **(E)** Distribution of genes with a marked change in both m^6^A level and total transcript level in *Alkbh5* overexpression cells compared to that in control cells. Alkbh5, AlkB homolog H5; GO, Gene Ontology; KEGG, Kyoto Encyclopedia of Genes and Genomes; m^6^A, N6-methyladenosine; 5′-UTR, 5′ untranslated region; m^6^A-seq, m^6^A sequencing; RNA-seq, RNA sequencing.

To further evaluate the role of *Alkbh5* in the differentiation of mDPC6T cells, RNA-seq and m^6^A-seq were performed using RNA from the *Alkbh5* overexpression group and negative control group undergoing odontoblast induction for 2 weeks. The differentially expressed genes following *Alkbh5* overexpression at Log2 ratio >1 or < −1 are shown in the heatmap (Figure 4A). Gene Ontology (GO) enrichment and Kyoto Encyclopedia of Genes and Genomes (KEGG) pathway analyses were performed to identify the biological processes and pathways that differed between the *Alkbh5* overexpression group and the negative control group. The significant GO biological process terms for the *Alkbh5* overexpression group included cell adhesion, cell differentiation, positive regulation of cell proliferation, and positive regulation of gene expression (Figure 4B). The top 10 enriched pathways identified *via* KEGG pathway analysis are shown in Figure 4C. The PI3K/AKT pathway was the most enriched pathway.

GO and KEGG enrichment analyses of upregulated or downregulated genes in the *Alkbh5* overexpression group are shown in Supplementary Figures S4A–D. Consistent with previous m^6^A-seq results, the m^6^A locus was highly enriched for typical RRACH motif in both the *Alkbh5* overexpression and negative control groups (Figure 4D). These m^6^A modifications were mainly situated in the exons and the 3′-untranslated region (Figure 4D). Based on the m^6^A-seq results, the total m^6^A peak density of mRNA was analyzed which showed that m^6^A peaks were abundantly present near those of stop codons (Supplementary Figure S4E). By incorporating transcriptome and m^6^A methylome analyses, the levels of 131 mRNA transcripts containing hypomethylated m^6^A peaks were significantly increased following *Alkbh5* overexpression (H-D), accounting for 50% of the 259 hypomethylated m^6^A genes (Figure 4E).

### *Alkbh5* Overexpression Enhanced *Runx2* mRNA Stability and Controlled the Activation of the PI3K/AKT Pathway to Modulate the Odontoblast Differentiation of mDPC6T Cells

**FIGURE 5:**
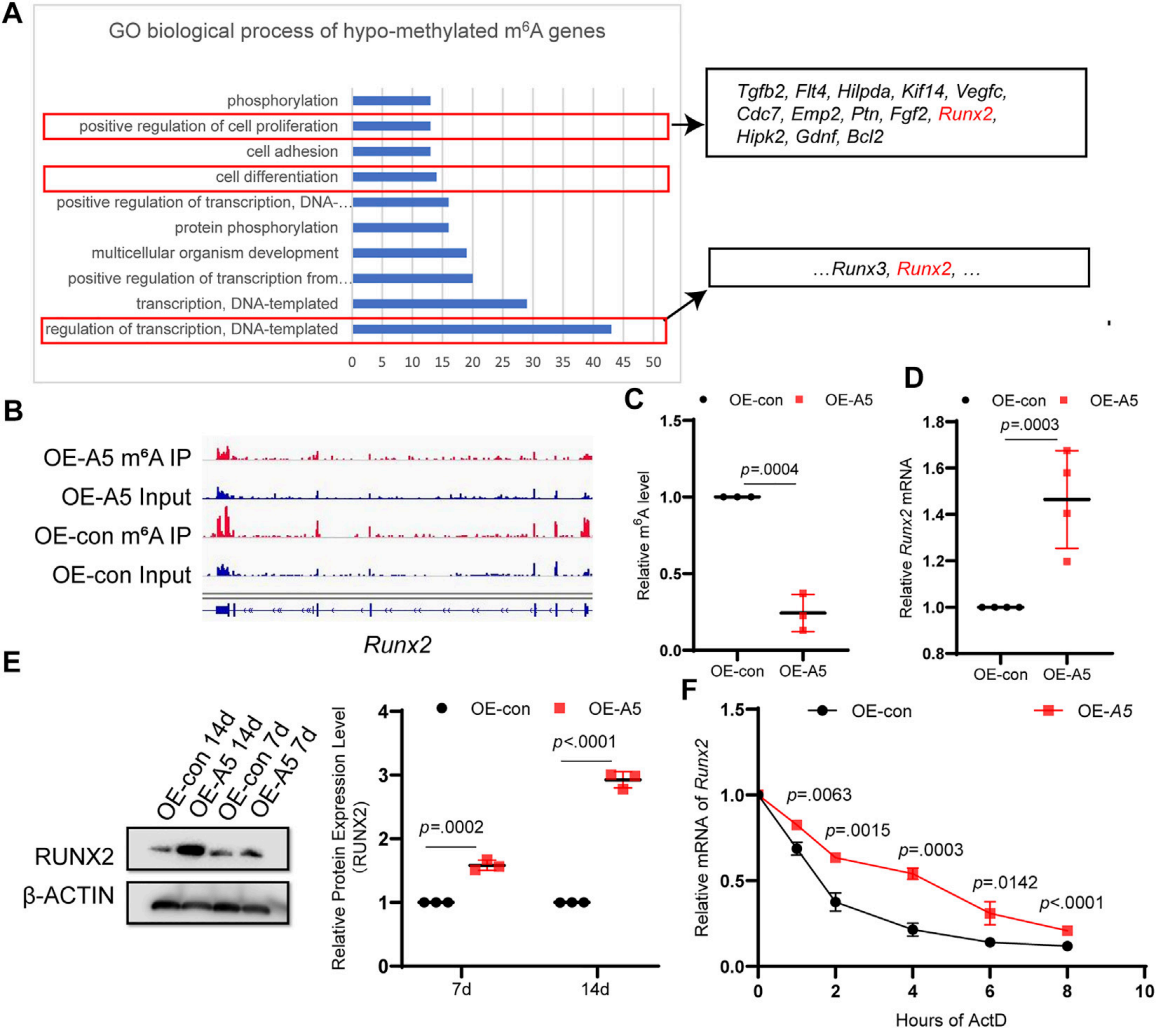
ALKBH5 modulated the stability of *Runx2* in an m^6^A-dependent manner. **(A)** GO enrichment analysis of differentially expressed genes in the hypomethylated m^6^A group, which included *Runx2*. **(B)** The m^6^A peaks of *Runx2* were analyzed using Integrative Genomics Viewer software. **(C)** m^6^A-IP-qPCR analysis of *Runx2* mRNA m^6^A modification in the *Alkbh5* overexpression and control groups. **(D)** *Runx2* mRNA expression level was measured using qPCR. **(E)** RUNX2 protein expression level was determined using western blotting. **(F)** The RNA stability of *Runx2* was determining by detecting the transcript abundance in mDPC6T cells transfected with negative or overexpressing lentivirus targeting *Alkbh5* after the addition of actinomycin **(D)**. Alkbh5, AlkB homolog H5; GO, Gene Ontology; Runx2, Runt-related transcription factor 2; m^6^A, N6-methyladenosine; qPCR, quantitative polymerase chain reaction.

*Alkbh5* overexpression reduces the methylation modifications on RNA (Jia et al., 2013; Zhang et al., 2016). GO enrichment analysis of hypomethylated m^6^A genes indicated that a number of genes were involved in positive regulation of transcription, cell differentiation, and positive regulation of cell proliferation (Figure 5A). RUNX2, which contributes to odontoblast differentiation and mineral accumulation, is involved in the aforementioned biological processes (Wen et al., 2020; Lin et al., 2021; Xiao et al., 2021). By comparing the peak calling via Integrative Genomics Viewer analysis, we observed that *Runx2* was modified by m^6^A, and the m^6^A peak in the *Alkbh5* overexpression group declined compared with that in the negative control group (Figure 5B). To determine whether *Runx2* was regulated by Alkbh5, we found that m^6^A-specific antibodies significantly enriched *Runx2*, which is in accordance with the m^6^A-seq results based on methylated m^6^A-qRT-PCR experiments; the enrichment of m^6^A in *Runx2* was lower than that in the negative control group after *Alkbh5* overexpression (Figure 5C). *Alkbh5* overexpression upregulated *Runx2* during odontoblast differentiation of mDPC6T cells for seven or 14 days (Figures 5D,E). *siAlkbh5* downregulated *Runx2* during odontoblast differentiation of mDPC6T cells for 3 days (Supplementary Figures S5A, B). These results confirmed that Alkbh5 regulated the methylation level of *Runx2* mRNA. Subsequently, we explored the mechanisms underlying the specific Alkbh5-mediated regulation of *Runx2*. mDPC6T cells transfected with *Alkbh5-*overexpressing lentivirus or negative lentivirus were cultured with a transcription inhibitor actinomycin D. The remaining amounts of *Runx2* mRNA were collected. A higher level of *Runx2* mRNA was retained in the *Alkbh5* overexpression group than that in the negative group, indicating a prolonged half-life of *Runx2* mRNA in the *Alkbh5* overexpression group (Figure 5F). A lack of stable *Runx2* mRNA led to a decrease in Runx2 protein expression; Alkbh5 modulated the expression of *Runx2* by affecting the rate of mRNA degradation *in vitro*.

**FIGURE 6:**
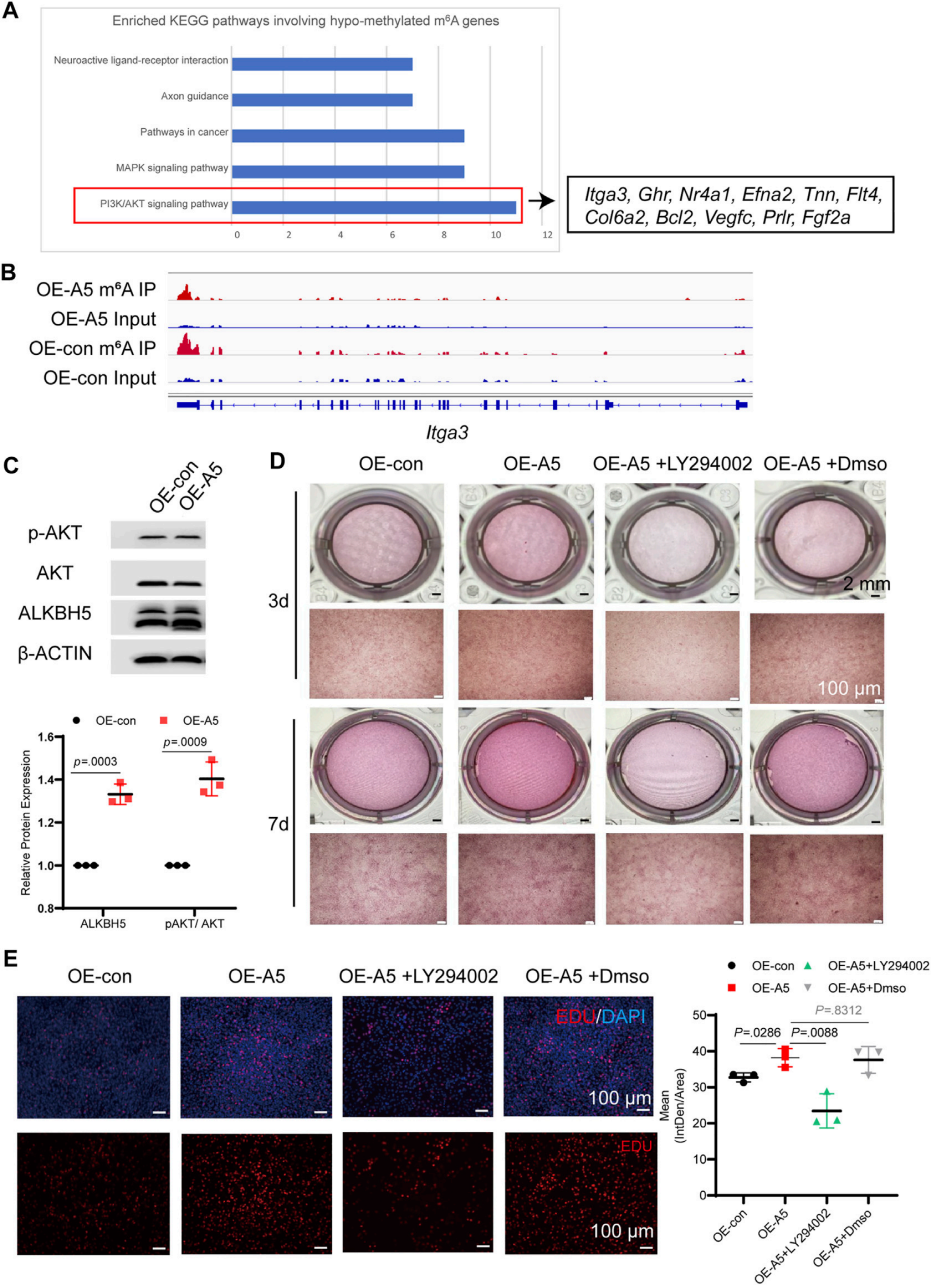
ALKBH5 activated the AKT signaling pathway to regulate odontoblast differentiation in mDPC6T cells. **(A)** KEGG pathway analysis of hypomethylated m^6^A differentially expressed genes and genes associated with the PI3K/AKT pathway. **(B)** The m^6^A peaks of integrin alpha-3 were analyzed using Integrated Genomics Viewer software. **(C)** The total AKT and p-AKT levels were measured in *Alkbh5-*overexpressing and control mDPC6T cells *via* western blotting. **(D)** ARS assay after adding LY294002 to *Alkbh5-*overexpressing cells. The scale bars: 2 mm and 100 μm (high magnification). **(E)** Immunofluorescence staining of EdU in mDPC6T cells and its quantitative analyses (*n* = 6). Scale bar: 100 μm Alkbh5, AlkB homolog H5; KEGG, Kyoto Encyclopedia of Genes and Genomes; PI3K, Phosphoinositide 3-kinase; AKT, protein kinase B; p-AKT, phosphorylated AKT; m^6^A, N6-methyladenosine.

We performed KEGG analysis for the hypomethylated m^6^A genes (Figure 6A). The enrichment analyses showed that the Alkbh5-altered genes were clustered in the PI3K/AKT pathway, mitogen-activated protein kinase signaling pathway, and pathways in cancer. Pathway enrichment analyses indicated that ALKBH5 played a role in the regulation of the PI3K/AKT pathway during odontoblast differentiation of mDPC6T cells, which was consistent with RNA-seq results (Figure 4C). The peak calling of related genes in the PI3K/AKT pathway suggested that integrin alpha-3*,* growth hormone receptor, Fms-related tyrosine kinase 4*,* collagen alpha-2(VI), Bcl2 apoptosis regulator, and prolactin receptor were modified by m^6^A, and the m^6^A peak in the *Alkbh5* overexpression group was lower than that in the negative group (Figure 6B, Supplementary Figures S4F, G). To confirm whether Alkbh5 regulated odontoblast differentiation of mDPC6T cells via the PI3K/AKT pathway, the phosphorylation level of AKT was evaluated. *Alkbh*5 overexpression was associated with an increase in AKT phosphorylation level compared with that in the control group (Figure 6C). *Alkbh5*-overexpressing cells were treated with the PI3K/AKT pathway inhibitor LY294002 to observe the effects on calcium nodules and the proliferation of mDPC6T cells during odontoblast differentiation. LY294002 treatment in combination with *Alkbh5* overexpression decreased the number of calcium nodules and proliferation of mDPC6T cells to the level observed in the negative control group (Figure 6D). DNA replication activity can be directly and accurately detected based on the specific reaction of fluorescent dyes with EdU. Edu cell proliferation assay showed that LY294002 treatment in combination with *Alkbh5* overexpression restrain the proliferation of mDPC6T cells to the level observed in the negative control group (Figure 6E). Meanwhile, si*Alkbh*5 was associated with a decrease in AKT phosphorylation level compared with that in the control group (Supplementary Figures S5C, D). *siAlkbh5* cells were treated with LY294002 or the PI3K/AKT pathway activator SC79 to observe the effects on calcium nodules of mDPC6T cells during odontoblast differentiation. LY294002 treatment in combination with *siAlkbh5* decreased the number of calcium nodules and SC79 treatment in combination with *siAlkbh5* increased the number of calcium nodules (Supplementary Figure S5E). Therefore, *Alkbh5* overexpression enhanced the PI3K/AKT pathway activity and mDPC6T proliferation during odontoblast differentiation *in vitro*.

### Impaired Tertiary Dentin Formation in *Alkbh5-cKO* Mice

**FIGURE 7:**
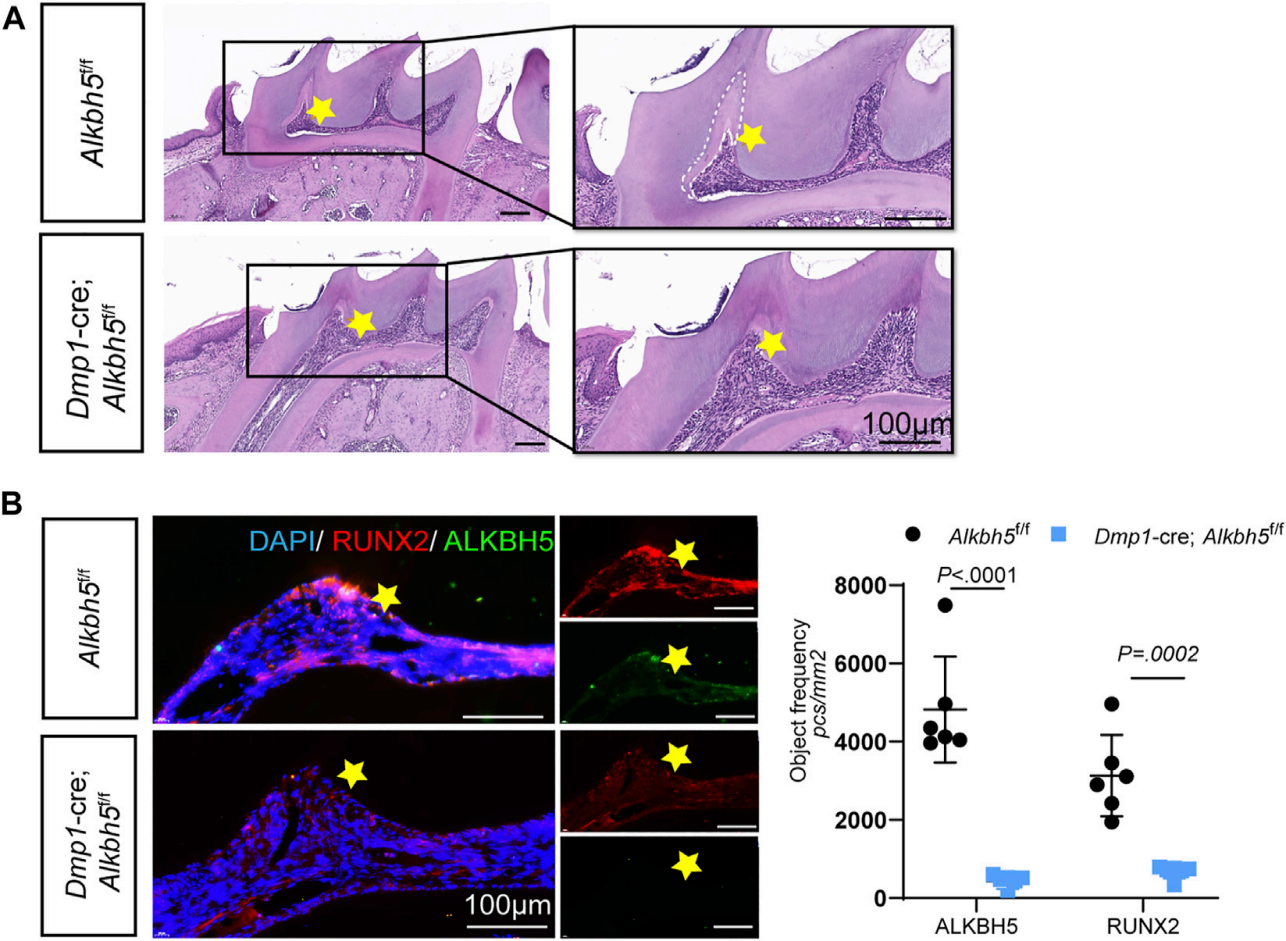
Impaired tertiary dentin formation in *Dmp1*-Cre; *Alkbh5* ^*fl/fl*^ mice. **(A)** Representative H&E staining of the maxillary first molar structure in the *Dmp1*-Cre; *Alkbh5* ^fl/fl^ and *Alkbh5* ^fl/fl^ mice after drilling for 14 days. The formation of tertiary dentin formation occurred in the proximal mesial pulp chamber near the defect. Tertiary dentin formation was inconspicuous in *Dmp1*-Cre; *Alkbh5* ^fl/fl^ mice. Asterisks (*) indicate the pulp horn and dotted lines indicate the boundaries between primary dentin and tertiary dentin. **(B)** The expression of ALKBH5 and RUNX2 in maxillary first molars was detected *via* immunofluorescence. Quantitative analyses of the expression of ALKBH5 and RUNX2 in *Dmp1*-Cre; *Alkbh5* ^fl/fl^ and *Alkbh5* ^fl/fl^ mice. Scale bar: 100 μm Alkbh5, AlkB homolog H5; H&E, hematoxylin and eosin.

Tertiary dentin formation involves the production of a tertiary dentine matrix by viable odontoblast cells or a new generation of odontoblast-like cells in response to an appropriate stimulation (Smith et al., 1995). To further investigate the role of *Alkbh5* in tertiary dentin formation, a cavity was placed on the proximal mesial surface of the maxillary first molar of mice (black dotted circle; Supplementary Figure S6A). After 2 weeks of cavity preparation, *Alkbh5* ^fl/fl^ mice showed tertiary dentin formation at the mesial side of the pulp chamber, while *Alkbh5-cKO* mice showed decreased tertiary dentin formation (Figure 7A). Based on our previous results on the deletion of *Alkbh5* in terminal odontoblasts that affected primary dentinogenesis by regulating proliferation and apoptosis in terminal odontoblasts*,* we investigated whether *Alkbh5* expression could affect tertiary dentin formation *via* odontoblast proliferation (Figures 3F–I). Double-staining of Alkbh5 and KI67 in the maxillary first molar were performed after cavity preparation. KI67 and Alkbh5 were expressed in the pre-odontoblasts near tertiary dentin in Alkbh5^fl/fl^ mice; weak signals of KI67 were found in *Alkbh5-cKO* mouse samples (Supplementary Figure S6B).

During dentinogenesis, RUNX2 plays a role in epithelial–mesenchymal interactions and is closely related to the formation of calcified tooth tissue (D'Souza et al., 1999; Wen et al., 2020). Based on our previous results, *Runx2* was upregulated during odontoblast differentiation of mDPC6T cells and the overexpression of *Alkbh5* prolonged the half-life of *Runx2* transcripts *in vitro* (Figures 5D–F). Therefore, we investigated whether *Alkbh5* could stimulate odontoblasts to produce tertiary dentin *via Runx2*. Double staining of Alkbh5 and Runx2 in the maxillary first molar were performed after cavity preparation. Strong signals indicated that Runx2 and Alkbh5 were expressed in the pre-odontoblasts near tertiary dentin in *Alkbh5* ^fl/fl^ mice; however, weaker positive signals for Runx2 and Alkbh5 were observed in *Alkbh5-cKO* mice (Figure 7B). Therefore, *Alkbh5* may promote tertiary dentin formation *via* Runx2.

## Discussion

Several studies have demonstrated that dentinogenesis is regulated by multiple genetic factors. Epigenetic mechanisms regulating odontoblast differentiation are of great interest (Bae et al., 2018; Li S. et al., 2018; Zhao et al., 2018; Wen et al., 2020; Yu and Klein, 2020). m^6^A, an epitranscriptomic modification, is regulated by methyltransferases and demethylases (Jia et al., 2013; Punekar et al., 2013; Wang et al., 2016; Zhang et al., 2016). The function of RNA m^6^A methyltransferase METTL3 is essential in tooth root development (Sheng et al., 2021). However, the mechanism by which ALKBH5 is controlled as a demethylase during odontoblast differentiation and dentinogenesis is unclear. Here, we demonstrated that *Alkbh5* was upregulated during mouse odontoblast differentiation. *Alkbh5* deletion in odontoblasts reduced the number of odontoblasts and increased the thickness of pre-dentin in mice. Further investigation revealed that Alkbh5 prolonged the half-life of Runx2 transcripts in an m^6^A-dependent manner and influenced the activity of the PI3K/AKT pathway to regulate mDPC6T proliferation and differentiation. Moreover, we verified that Alkbh5 promoted tertiary dentin formation *via* Runx2.

m^6^A is involved in several biological processes and regulates pre-mRNA splicing, mRNA decay, and translation (Jia et al., 2013; Punekar et al., 2013; Wang X. et al., 2014; Zhang et al., 2016). This modification has been demonstrated to modulate the maintenance of pluripotency and differentiation of embryonic stem cells, as well as reprogramming of somatic cells (Wang Y. et al., 2014). ALKBH5 is a well-known m^6^A demethylase which has extensive involvement in important biological processes (Geula et al., 2015; Zhang et al., 2016). Recently, the roles of ALKBH5 in various biological processes have been confirmed, such as cell proliferation, tumor invasion and metastasis, and osteogenesis (Shen et al., 2020; Wang et al., 2020; Feng et al., 2021; Li et al., 2021). Therefore, the current study investigated the role of ALKBH5 in the regulation of odontoblast differentiation by evaluating odontoblast differentiation in mDPC6T cells and in a mouse conditional *Alkbh5* knockout model.

Immunohistochemistry analysis revealed that *Alkbh5* was expressed in functional odontoblasts *in vivo*. The m^6^A methyltransferase *Mettl3* and demethylase *Alkbh5* were evaluated in mDPC6T cells, which were cultured using odontoblast induction medium. Similar studies on the function of METTL3 in tooth development have been performed, METTL3 knockdown downregulates nuclear factor I C and inhibits odontogenesis in molar roots (Sheng et al., 2021). We focused on the role of RNA demethylase ALKBH5 in the differentiation of odontoblasts. The increased expression of *Alkbh5* was in agreement with the expression of the odontoblast markers *Dmp1* and *Dspp*. In addition, *Alkbh*5 overexpression upregulated *Dspp* and *Dmp1*. Mineralized nodule formation also increased after *Alkbh5* overexpression. Further, knockdown of *Alkbh5* inhibited the upregulation of odontoblast-related genes and reduced the mineral nodule formation.

DMP1 plays an important role in both dentin and bone mineralization (Staines et al., 2012). *Dmp1* expression is detected in cementoblasts, osteoblasts, and osteocytes and odontoblasts with secretory functions (Vijaykumar et al., 2019). In a conditional knocked out genetic animal model, the knockdown of *Dmp1* prevents the conversion of pre-dentin to dentin (Balic and Mina, 2011; Vijaykumar et al., 2019). Due to the stage-specific activation of Dmp1, the role of *Alkbh5* in odontoblasts with secretory function could be observed. *Alkbh5* could be deleted from terminal odontoblasts in *Alkbh5-cKO* mice. Alkbh5 deficiency has a mild effect on odontoblasts, but the accumulation resulting from this effect makes a difference at PN 28. After PN 28, there may be compensatory mechanisms *in vivo* to make up for this phenotype, and the specific reasons need further study.

Morphological analysis demonstrated that conditional knockout of *Alkbh5* led to a decrease in number of terminal odontoblasts and relatively thicker pre-dentin. Weaker positive signals for KI67 and stronger positive signals based on an *in situ* TUNEL assay using odontoblasts of *Dmp1*-Cre; *Alkbh5* ^fl/fl^ mice showed that conditional knockout of *Alkbh5* in the mandibular first molars affected the proliferation and apoptosis in terminal odontoblasts and subsequently altered the counts of terminal odontoblasts, which led to the decreased production and mineralization of pre-dentin. Therefore, *Alkbh5* promoted odontoblast differentiation and impaired primary dentin formation in *Alkbh5-cKO* mice.

To date, several studies have reported a strong relevance of epigenetic regulation to stem cells in tooth formation, and high-throughput sequencing technology can assist in studies on epigenetic regulation in tooth formation (Wang et al., 2013; Balic and Thesleff, 2015; Bae et al., 2018; Bakhit et al., 2018; Li G. et al., 2018; Zhao et al., 2018; Tao et al., 2019; Lin et al., 2021). To further evaluate the influence of *Alkbh5* on odontoblast differentiation, we performed RNA-seq and m^6^A-seq. By integrating transcriptome and m^6^A methylome analyses, GO enrichment analysis showed that various genes were enriched in regulation of transcription, cell differentiation, and positive regulation of cell proliferation and phosphorylation. Runx2 was necessary for tooth formation and was strongly related to the development of calcified hard tissues of teeth. Therefore, we investigated whether ALKBH5 could function as a downstream regulator of odontoblast differentiation *via Runx2*. Mechanistically, ALKBH5-m^6^A regulated the *Runx2* level by affecting the *Runx2* degradation rate. In contrast, KEGG analysis on hypomethylated m^6^A genes indicated that the PI3K/AKT pathway functions as a downstream signaling pathway. Recently, m^6^A methylation has been found to regulate PI3K/AKT pathway activity in diabetic kidney disease and endometrial cancer (Liu et al., 2018; Xu et al., 2021). This study found that the RNA demethylase ALKBH5 enhances PI3K/AKT pathway activity in mDPC6T cells to promote odontoblast proliferation and differentiation.

The dentin-pulp complex can mediate restorative changes after damage to the tooth (Yang et al., 2010; Park et al., 2020). Tertiary dentin formation can be induced by cavity preparation in mice (Saito et al., 2016; Saito et al., 2013). After cavity preparation, the differentiation of a new generation of odontoblast-like cells occurs from pulp stem cells, which can produce reactionary dentin (Balic and Thesleff, 2015; Vogel et al., 2016; Isono et al., 2021). Formation of tertiary dentin occurs near damaged dentin. We established a tertiary dentin formation model to evaluate the ability of odontoblasts to form tertiary dentin. Almost no tertiary dentin formation was observed in *Alkbh5-cKO* mice after cavity preparation. A larger number of KI67-positive cells were found in pre-odontoblasts near tertiary dentin in *Alkbh5* ^*fl/f*l^ mice than in *Alkbh5*-*cKO* mice.

During tooth development, Runx2 regulates dentinogenesis and mineralization (Gaikwad et al., 2001). Previous studies have demonstrated that RUNX2 is strongly expressed in pre-odontoblasts and the expression decreases in odontoblasts at E18.5 (Yamashiro et al., 2002; Chen S. et al., 2009; Chen Z. et al., 2009). However, RUNX2 is still expressed in pre-odontoblasts at PN 2.5 and has been demonstrated to enhance tooth development (Lin et al., 2021). In exception to skeletal defects in *Runx2*-deficient mice, the developing tooth germ is suppressed at the early development stages (Otto et al., 1997; D'Souza et al., 1999). Mechanistically, Runx2 regulates odontoblastic differentiation *via* activation of *Dspp* expression in mouse pre-odontoblast-like cells (Chen et al., 2005). In our experiments, *Alkbh5* was upregulated during odontoblast differentiation, and the expression pattern was consistent with the expression of *Runx2*. *Alkbh5* overexpression upregulated *Runx2*. A sustained increase in ALKBH5 leading to decreased m^6^A modification was necessary to balance the modification of m^6^A on *Runx2* mRNA under certain environments, which maintain the expression of Runx2. The formation of tertiary dentin with increased expression of *Runx2* can be detected in dental pulp after rat molar pulpotomy (Takeuchi et al., 2020). Runx2 is involved in the formation of dentin, including tertiary dentin. Further, Runx2 and Alkbh5 were expressed in odontoblasts near tertiary dentin in *Alkbh5* ^fl/fl^ mice suggesting that Alkbh5 enhanced the formation of tertiary dentin *via* Runx2 *in vivo*, which was consistent with our previous conclusion.

In conclusion, we found that *Alkbh5* was upregulated during odontoblast differentiation. A conditional knockout model was prepared to selectively knockout *Alkbh5* from odontoblasts cells to investigate the role of Alkbh5 in dentinogenesis. By combining RNA-seq and m^6^A-seq results, ALKBH5 was found to regulate *Runx2* by removing the m^6^A modification on mRNA and affecting its mRNA degradation. Simultaneously, ALKBH5 is involved in activating the PI3K/AKT pathway to promote odontoblast proliferation and differentiation of mDPC6T cells. In addition, the potential roles of ALKBH5 in promoting tertiary dentin formation were investigated by using a tertiary dentin formation model.

## Data Availability

The datasets presented in this study can be found in online repositories. The names of the repository/repositories and accession number(s) can be found below: https://ngdc.cncb.ac.cn/search/?dbId=gsa&q=CRA006723.

## Abbreviations

m^6^A, N6-methyladenosine; ALKBH5, AlkB homolog H5; mDPC6T, a mouse dental papilla cell line; Runx2, runt-related transcription factor 2; PI3K/AKT pathway, phosphatidylinositol 3-kinase pathway.
