# Supplementary figures and images for: Role of the Demethylase AlkB Homolog H5 in the Promotion of Dentinogenesis
Source: Front Physiol. 2022 Jun 15;13:923185. doi: 10.3389/fphys.2022.923185 (PMC9240783; doi:10.3389/fphys.2022.923185)

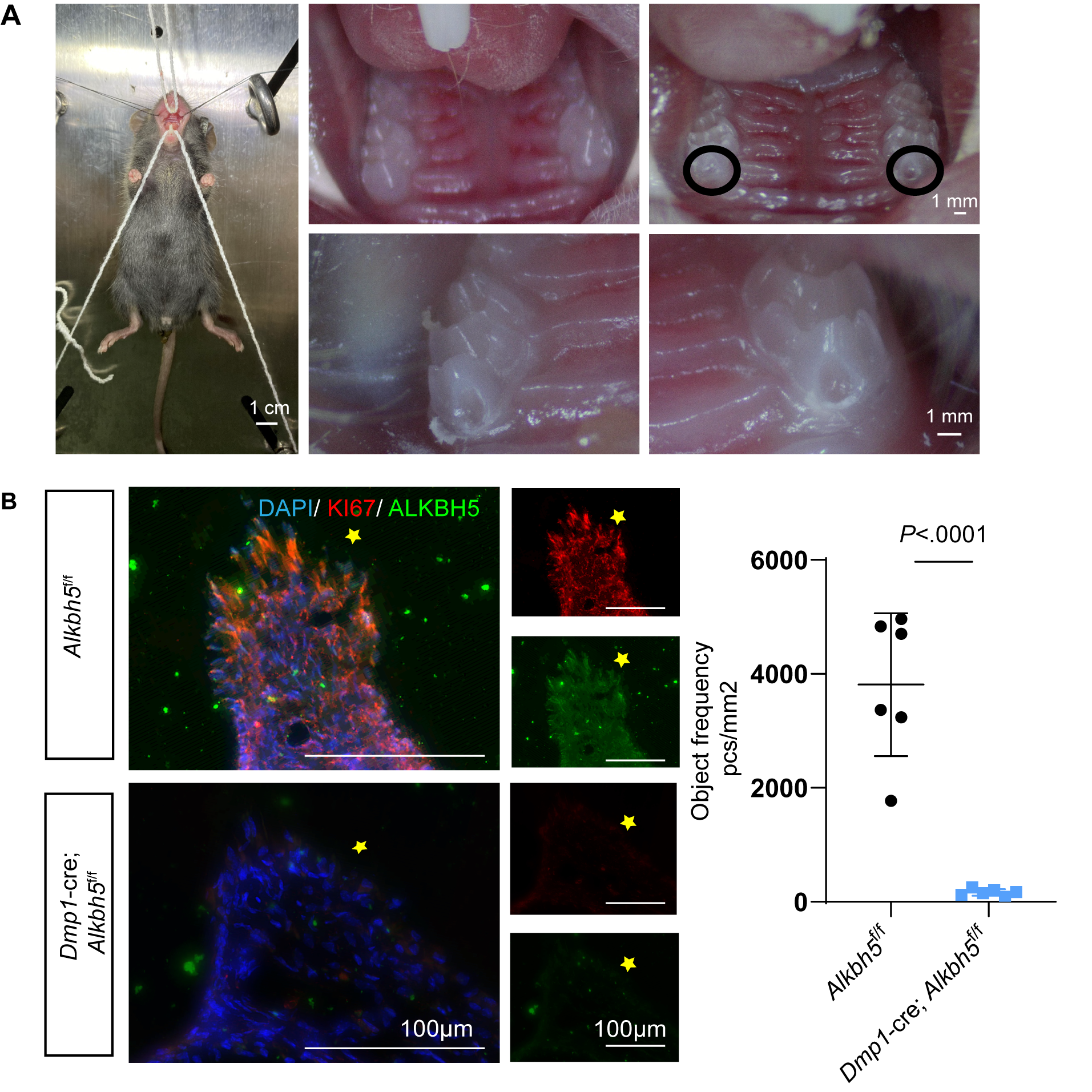

Supplement: Supplementary file 1 [file Image6.TIF]

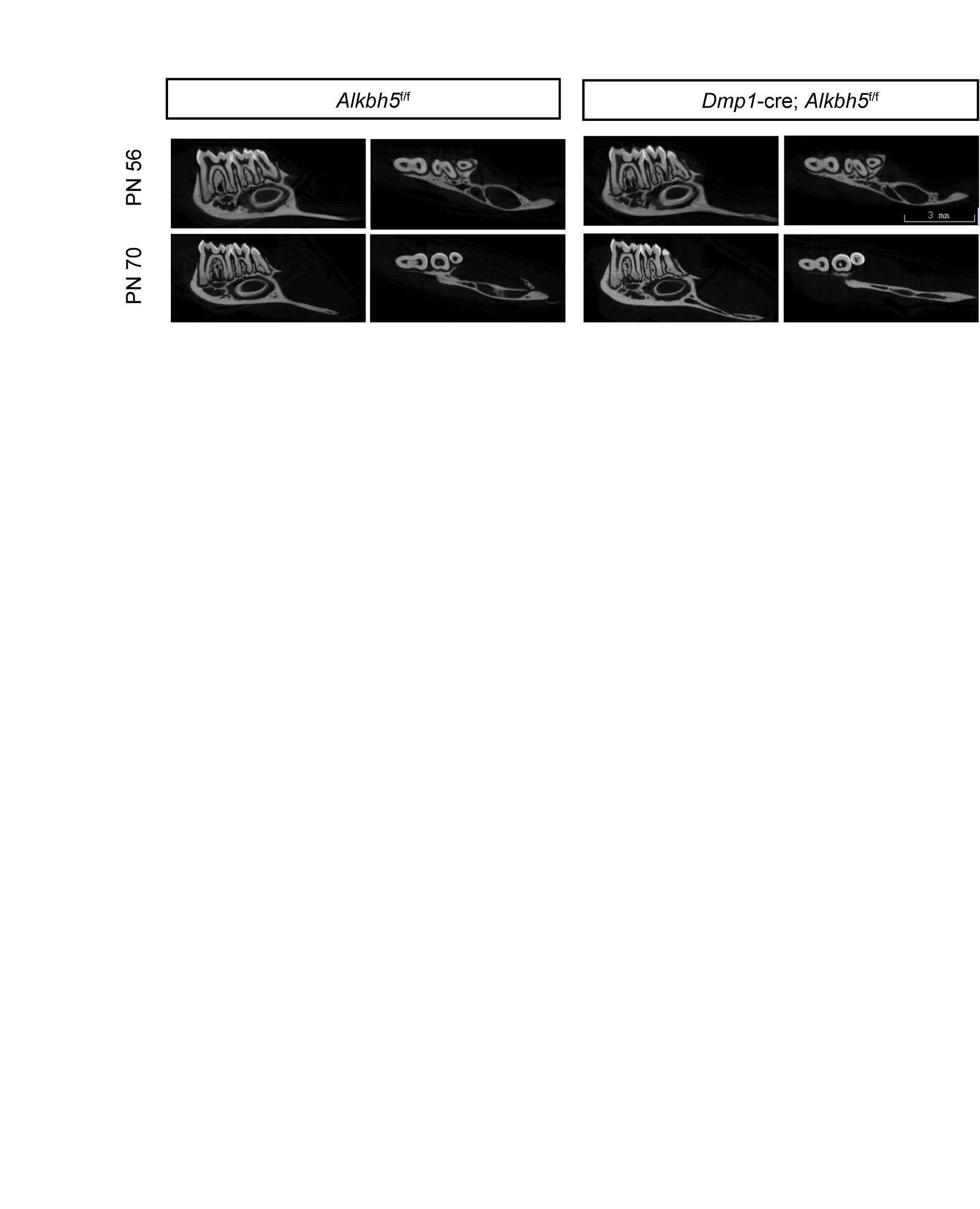

Supplement: Supplementary file 3 [file Image3.TIF]

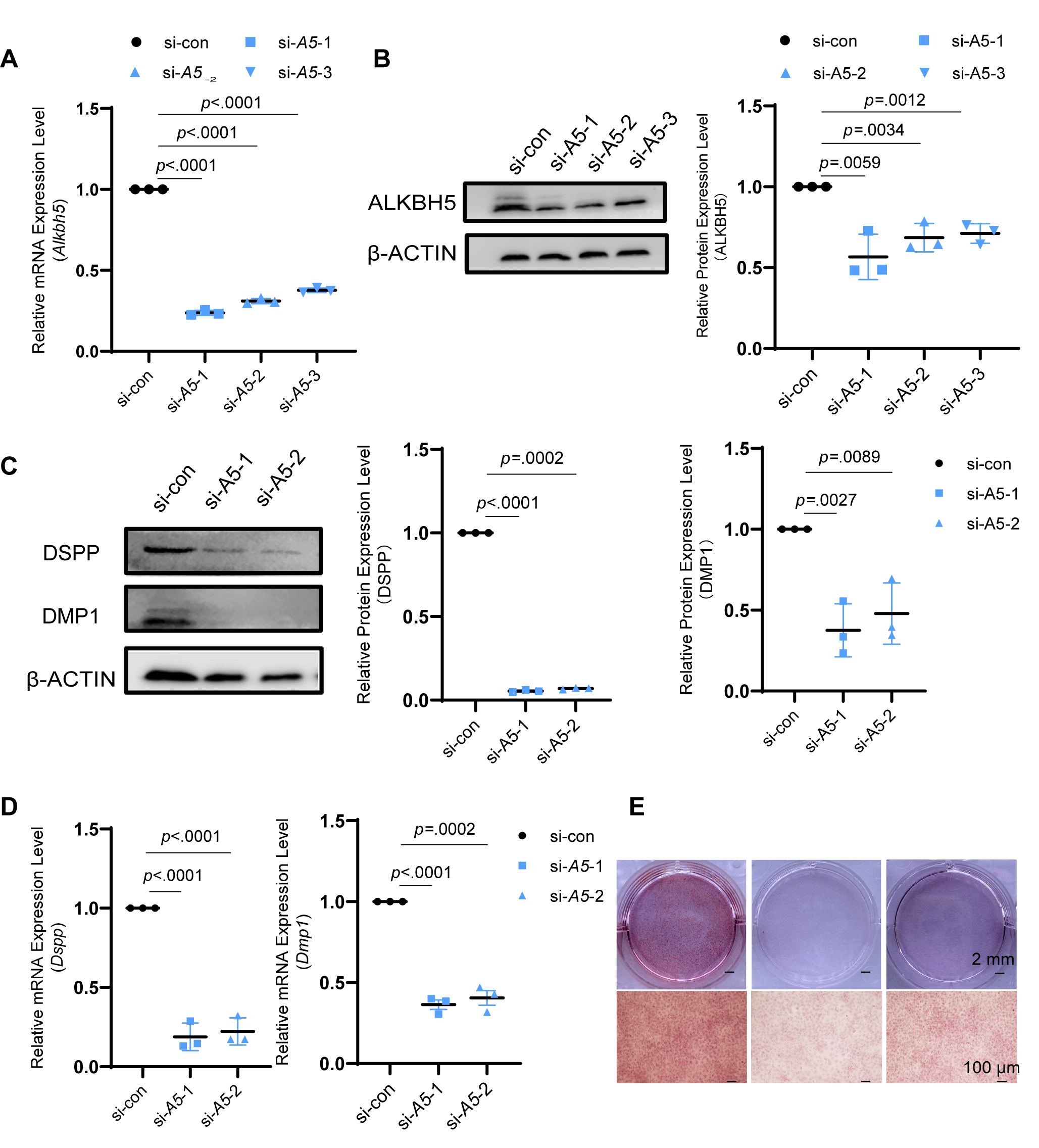

Supplement: Supplementary file 5 [file Image2.TIF]

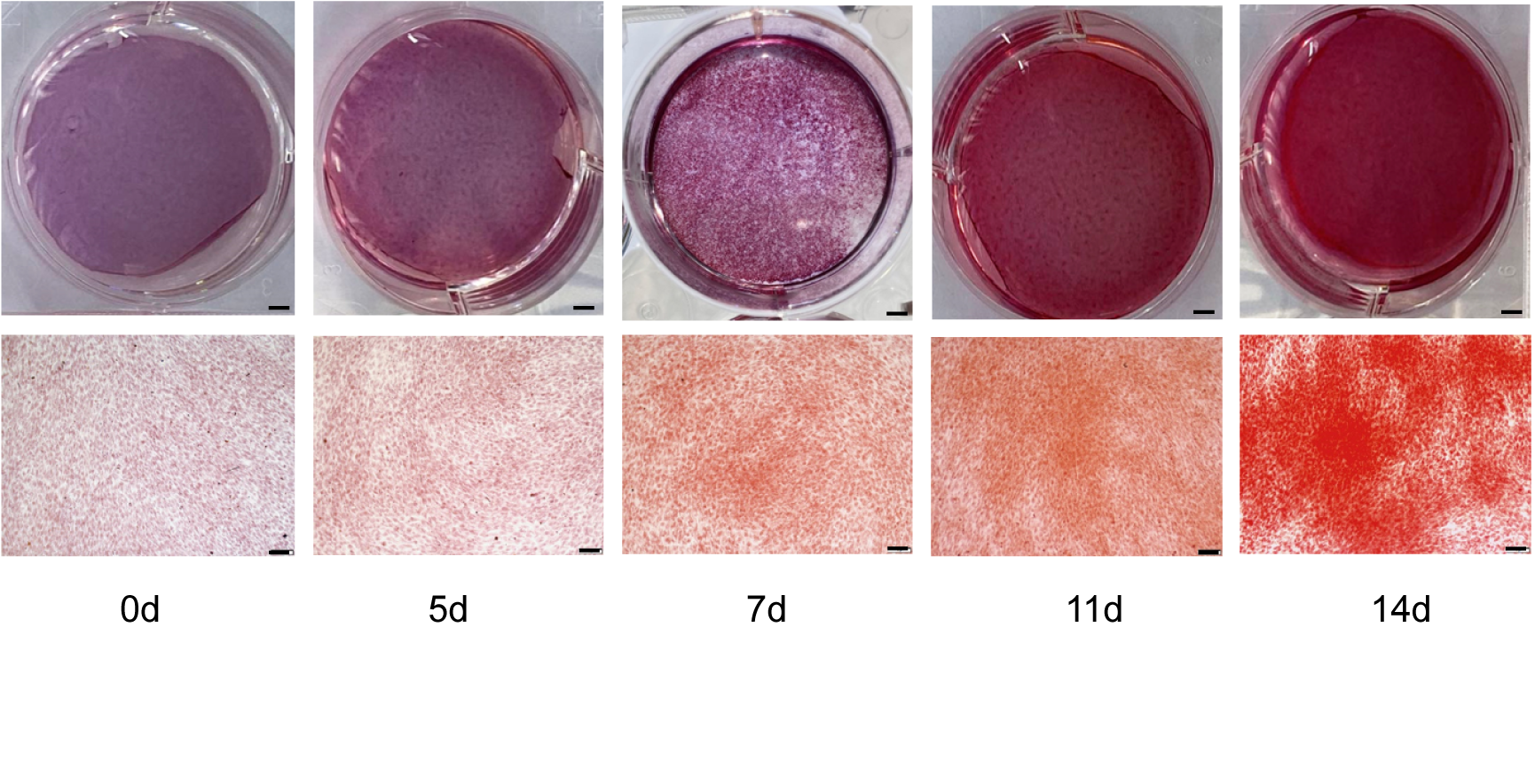

Supplement: Supplementary file 6 [file Image1.TIF]

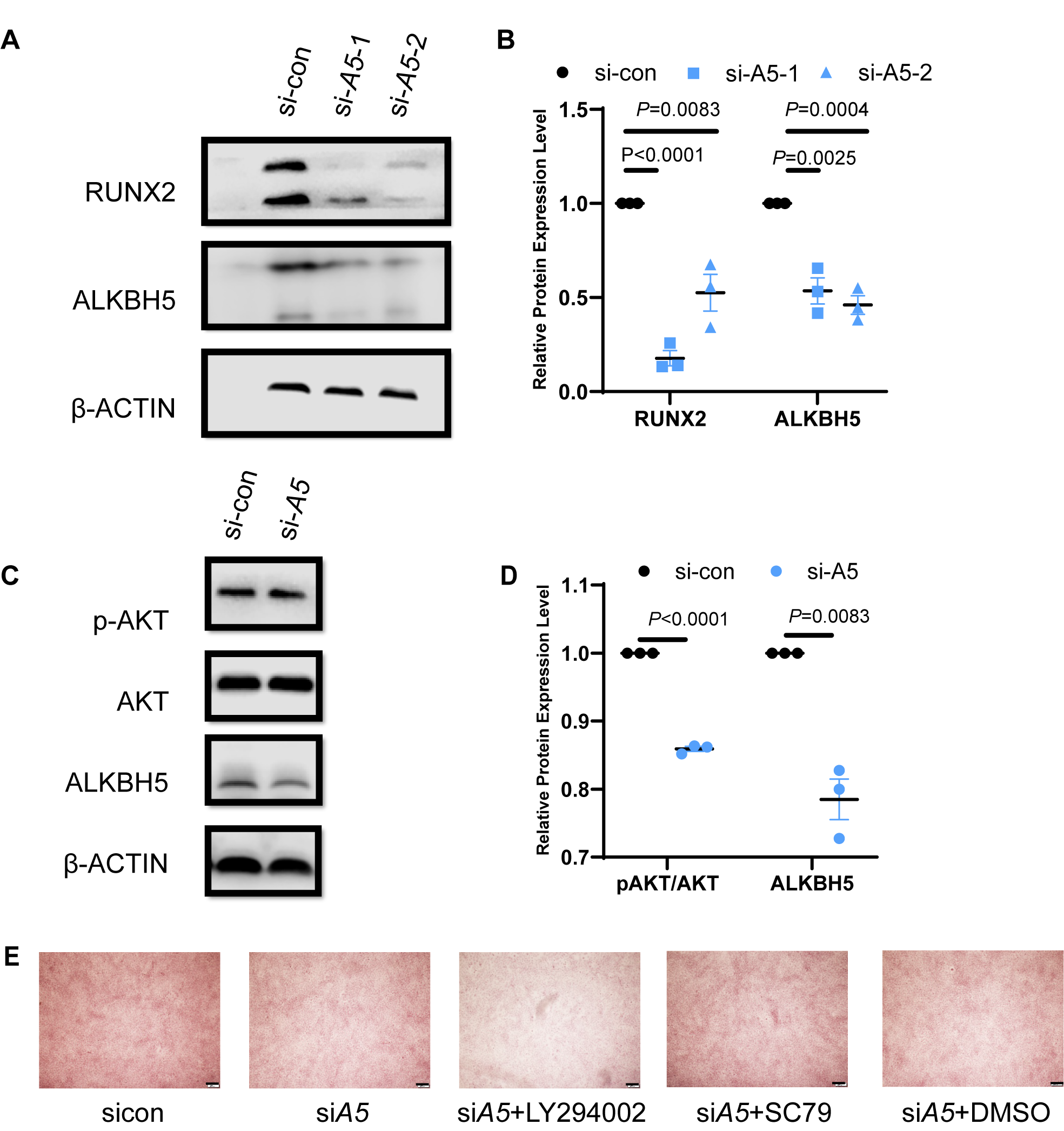

Supplement: Supplementary file 7 [file Image5.TIF]
