# Supplementary material for: Role of the Demethylase AlkB Homolog H5 in the Promotion of Dentinogenesis
Source: Front Physiol. 2022 Jun 15;13:923185. doi: 10.3389/fphys.2022.923185 (PMC9240783; doi:10.3389/fphys.2022.923185)
Supplement: Supplementary file 2 [file Table1.DOCX]

**Supplementary Figure**

**
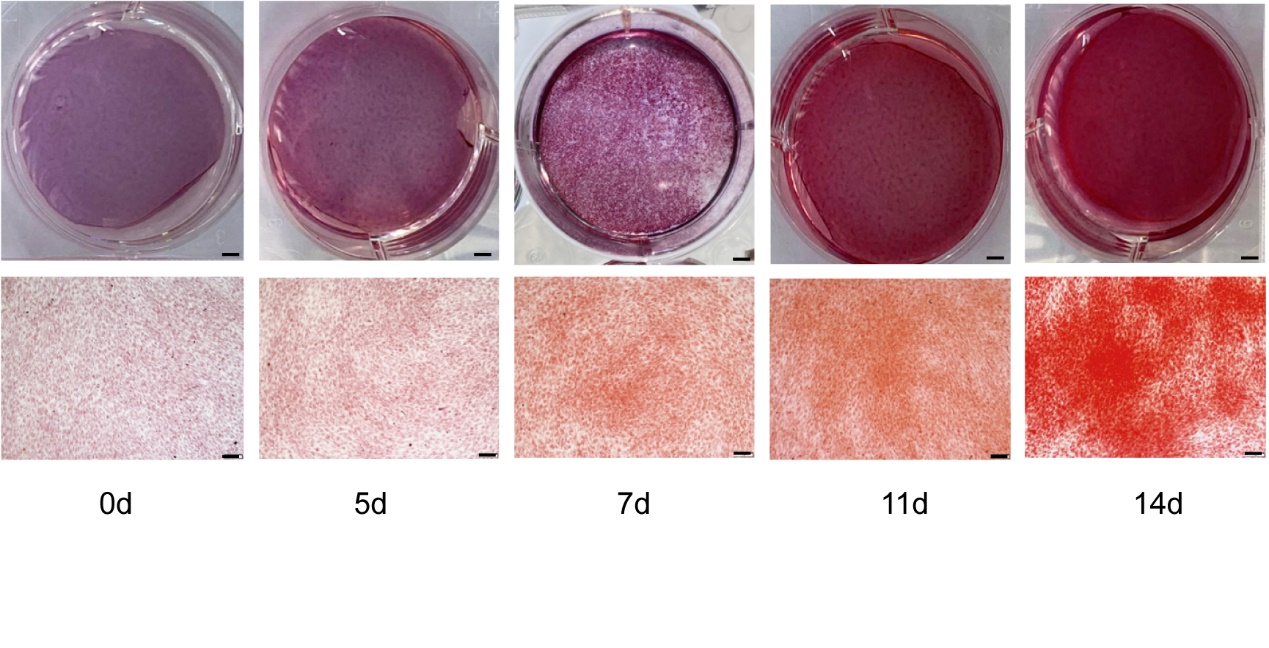
**

**Supplementary Figure 1. odontoblast differentiation of mDPC6T**

Mineralized nodules formation could be obvious in mDPC6T on days 0, 5, 7, 11, and 14 undergoing odontoblast differentiation. The scale bars represent 200 μm (original magnification ×100).


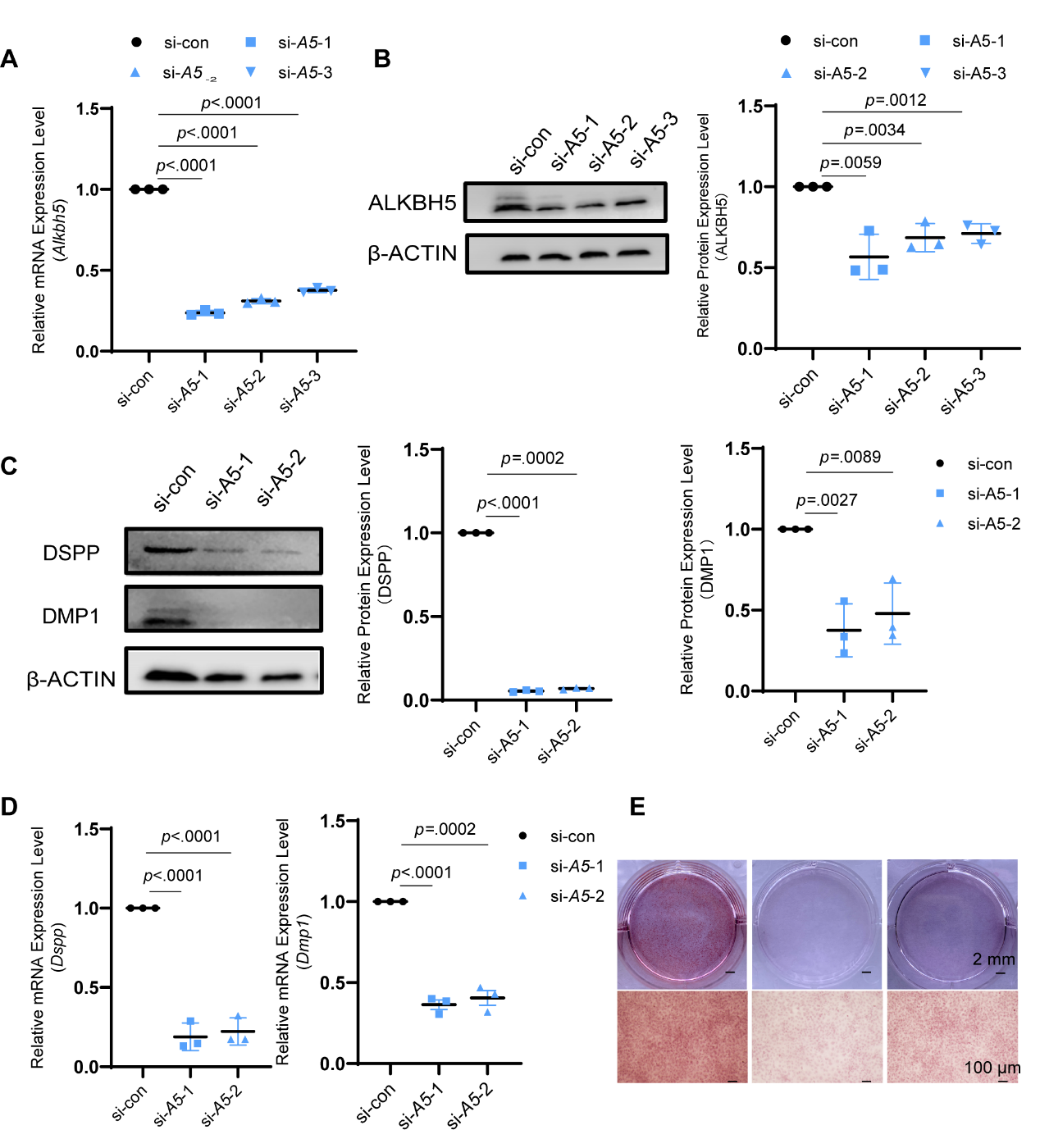


**Supplementary Figure 2. The effect of the siRNA-mediated depletion of *Alkbh5* expression in mDPC6T.**

(A) *Alkbh5* mRNA was measured in mDPC6T induced odontoblast differentiation following siRNA treatment by qRT-PCR.

(B) ALKBH5 protein expression was measured in mDPC6T induced odontoblast differentiation following siRNA treatment by western blot analysis.

(C) DSPP and DMP1 protein levels in the si*Alkbh5* group and sicon group were measured using western blot during 3 days odontoblast induction.

(D) *Dspp* and *Dmp1* mRNA expression in the si*Alkbh5* group and sicon group were measured using qRT-PCR during 3 days odontoblast induction.

(E) The formation of mineralized nodules was obvious in the si*Alkbh5* group and sicon group undergoing odontoblast induction on days 3. The scale bars: 2 mm and 100μm (high magnification).


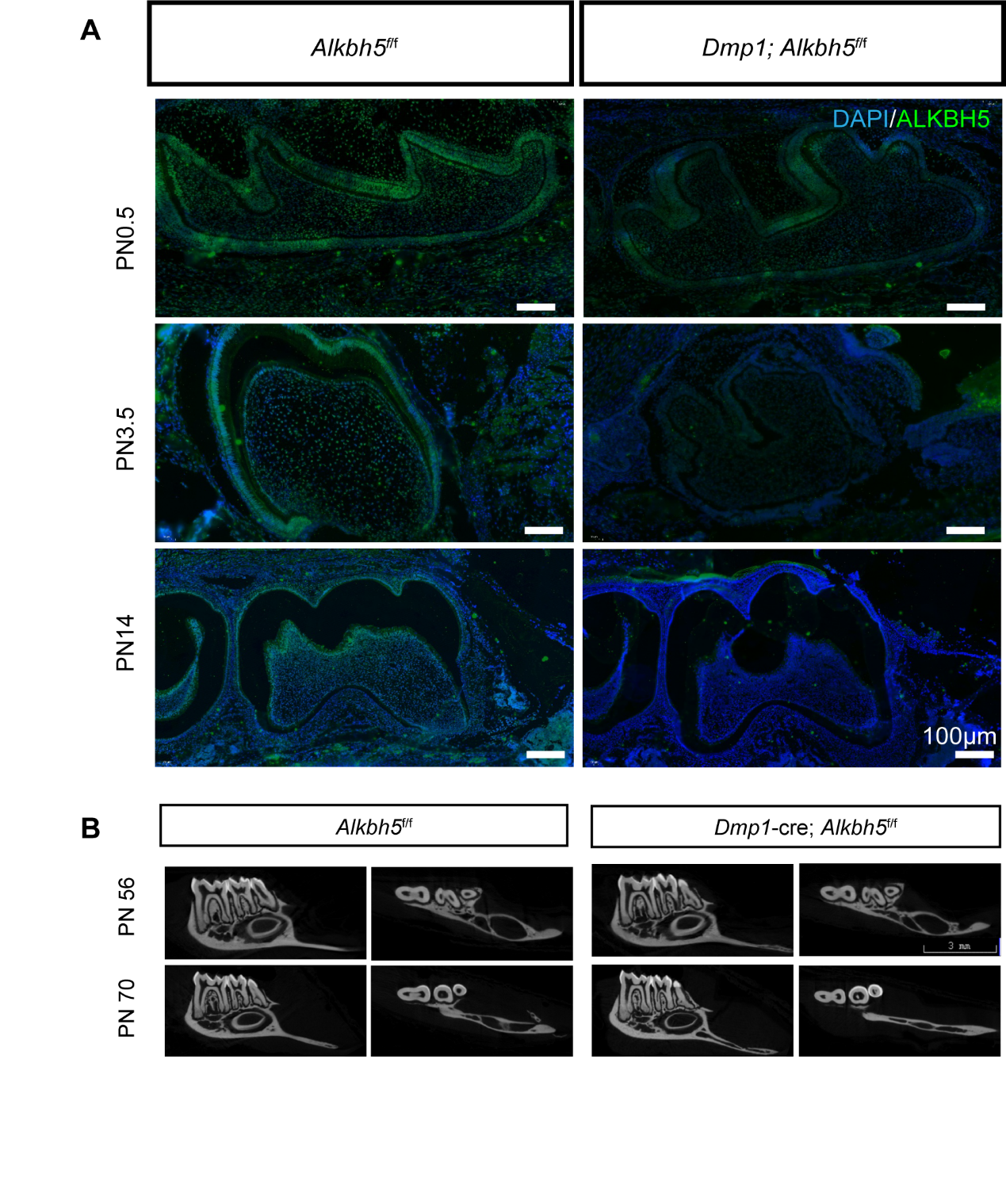


**Supplementary Figure 3. The expression of ALKBH5 protein was deleted in conditional knockout *Dmp1*-Cre; *Alkbh5^fl/fl^* mice.**

Computerized structural μCT imaging of the mandibular first molar from *Dmp1*-Cre; *Alkbh5^fl/fl^* and *Alkbh5^fl/fl^* mice at PN 56 and PN 70. (n= 6 mice). Scale bar: 3 mm


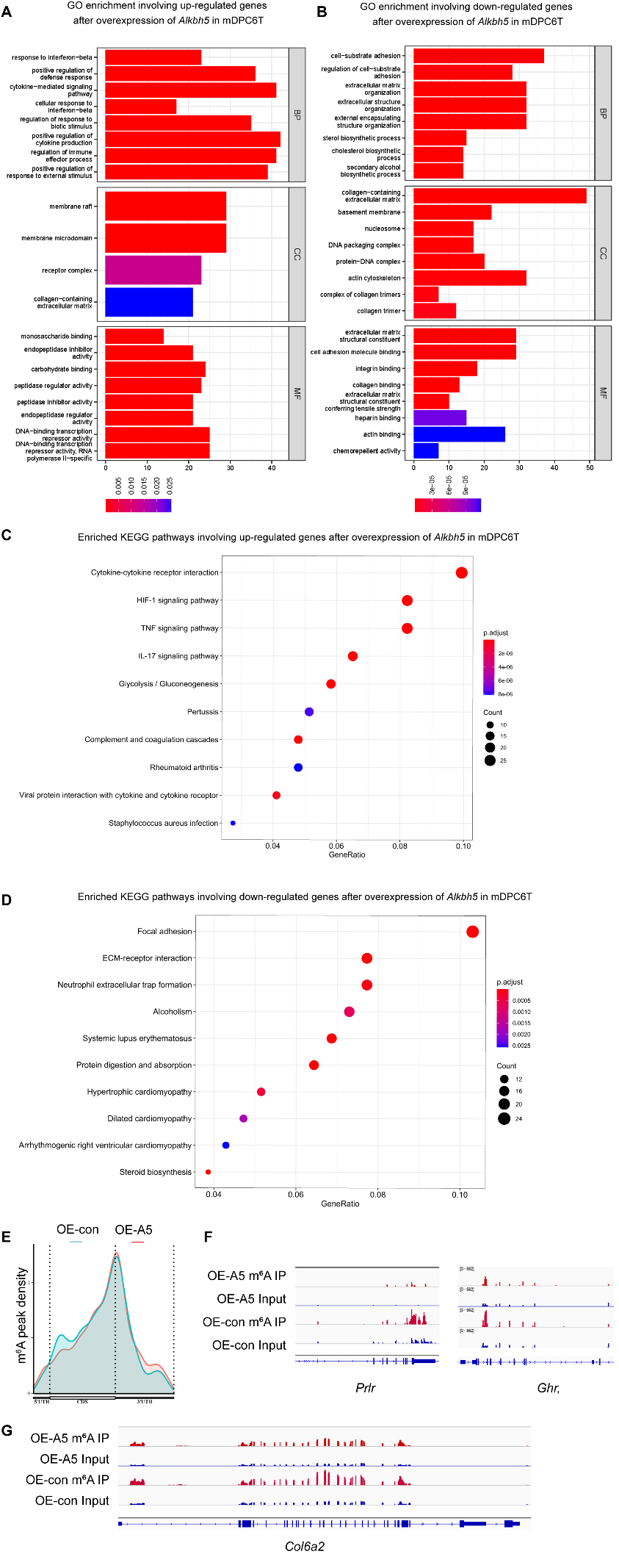


**Supplementary Figure 4. Whole-transcriptome m^6^A-seq and RNA-seq overexpression of ALKBH5 downstream regulatory genes.**

(A, B) GO analysis of up- or down-regulated genes by *Alkbh5* overexpression were shown.

(C, D) KEGG enrichment of up- or down-regulated genes by *Alkbh5* overexpression were shown.

(E) m^6^A peaks density distribution on mRNA transcripts.

(F, G) The m^6^A peaks of *Ghr, Flt4, Col6a2, Bcl2, Prlr* was analyzed by IGV software.


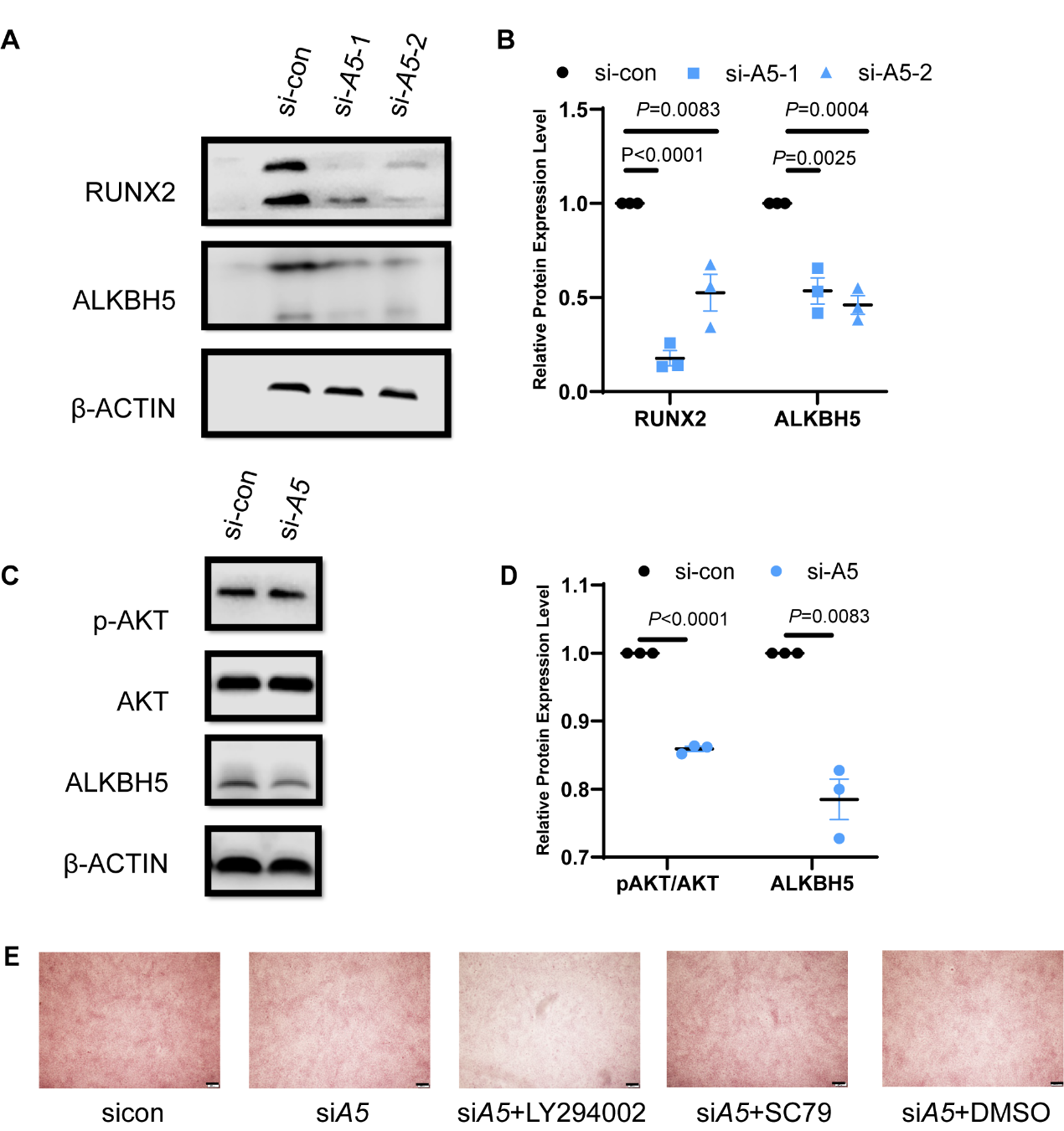


**Supplementary Figure 5. siRNA-mediated depletion of *Alkbh5* inhibited the AKT signaling pathway to regulate odontoblast differentiation in mDPC6T cells.**

1. The RUNX2 and ALKBH5 levels were decreased in mDPC6T cellsvia western blotting.
2. Quantification of the RUNX2 and ALKBH5 protein expression level in si*Alkbh5* and sicon mDPC6T cells during odontoblast induction.
3. The p-AKT and ALKBH5 levels were decreased in si*Alkbh5* mDPC6T cells via western blotting.
4. Quantification of the pAKT/AKT and ALKBH5 protein expression level in si*Alkbh5* and sicon mDPC6T cells during odontoblast induction.
5. ARS assay after adding LY294002 or SC79 to si*Alkbh5* cells. When the PI3K-AKT inhibitor LY294002 was added to the si*Alkbh5* mDPC6T cells, the mineralized nodules decreased; mineralized nodules increased when PI3K-AKT activator SC79 was added to si*Alkbh5* mDPC6T cells. The scale bars: 100 μm.


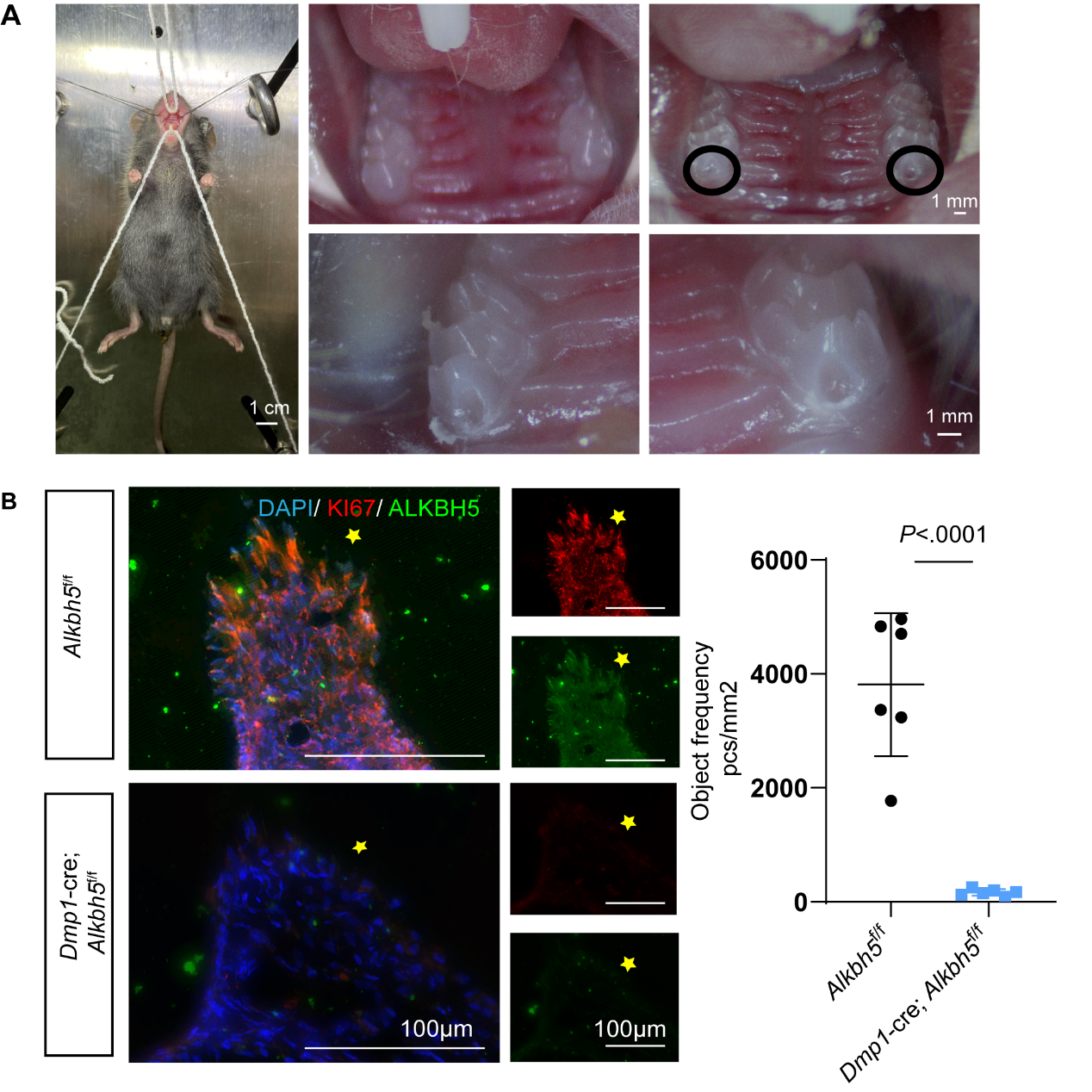


**Supplementary Figure 6. Diagram of the restoration of the dentin model.**

(A) Cavity preparation on the proximal mesial surface of the maxillary first molar (Black dotted circle).

(B) The expression of ALKBH5 and KI67 of the first maxillary molars was detected by immunofluorescence. Quantitative analyses of the expression of ALKBH5 and KI67 for *Dmp1*-Cre; *Alkbh5*^fl/fl^ and *Alkbh5*^fl/fl^ mice (n= 3 mice). Scale bar: 100μm.

**Supplementary Table 1**

**The siRNA sequences for *Alkbh5* knockdown.**

| M *Alkbh5* siRNA | Sense | Antisense |
| --- | --- | --- |
| Si-1 | CCACCCAGCUAUGCUUCAGAUTT | AUCUGAAGCAUAGCUGGGUGGTT |
| Si-2 | GAUCCUGGAAAUGGACAAAGATT | UCUUUGUCCAUUUCCAGGAUCTT |
| Si-3 | CCUAUGAGUCCUCGGAAGAUUTT | AAUCUUCCGAGGACUCAUAGGTT |

**Supplementary Table 2**

**Primers for qRT-PCR**

| Gene | Forward Primer | Reverse Primer |
| --- | --- | --- |
| *Dspp* | TCAGGAGCCAGGTAGGCATC | GGTCGCATCTCCATTCTGGC |
| *Dmp1* | CCCACGAGCACTCAGGATTC | AGAGACGTGGGACCTTCTGA |
| *Mettl3* | CCGTAGTGATAGTCCCGTGC | TAGTTCAGGGGCCACAGATG |
| *Alkbh5* | GCATACGGCCTCAGGACATTA | TTCCAATCGCGGTGCATCTAA |
| *Fto* | TTCATGCTGGATGACCTCAATG | GCCAACTGACAGCGTTCTAAG |
| *Runx2* | CCCTCGGAGAGGTACCAGAT | CTCGGCGGAGTAGTTCTCATC |
| *Gapdh* | GGTCATCCCAGAGCTGAACG | CCAAGTCACTGTCACACCAGA |
